# Cobaltabis(dicarbollide) ([*o*-COSAN]^−^) as Multifunctional Chemotherapeutics: A Prospective Application in Boron Neutron Capture Therapy (BNCT) for Glioblastoma

**DOI:** 10.3390/cancers13246367

**Published:** 2021-12-19

**Authors:** Miquel Nuez-Martinez, Catarina I. G. Pinto, Joana F. Guerreiro, Filipa Mendes, Fernanda Marques, Amanda Muñoz-Juan, Jewel Ann Maria Xavier, Anna Laromaine, Valeria Bitonto, Nicoletta Protti, Simonetta Geninatti Crich, Francesc Teixidor, Clara Viñas

**Affiliations:** 1 Institut de Ciència de Materials de Barcelona (ICMAB-CSIC), Campus UAB, 08193 Bellaterra, Spain; mnuez@icmab.es (M.N.-M.); amunoz@icmab.es (A.M.-J.); jxavier@icmab.es (J.A.M.X.); alaromaine@icmab.es (A.L.); teixidor@icmab.es (F.T.); 2 Centro de Ciências e Tecnologias Nucleares, Departamento de Engenharia e Ciências Nucleares, Instituto Superior Técnico, Universidade de Lisboa, 2695-066 Bobadela, Portugal; catarina.pinto@tecnico.ulisboa.pt (C.I.G.P.); joanaguerreiro@ctn.tecnico.ulisboa.pt (J.F.G.); fmendes@ctn.tecnico.ulisboa.pt (F.M.); fmarujo@ctn.tecnico.ulisboa.pt (F.M.); 3 Department of Molecular Biotechnology and Health Sciences, University of Torino, 10126 Torino, Italy; valeria.bitonto@unito.it (V.B.); simonetta.geninatti@unito.it (S.G.C.); 4 Department of Physics, University of Pavia, 27100 Pavia, Italy; nicoletta.protti@unipv.it

**Keywords:** boron clusters, metallacarboranes, small molecules, COSAN, boron neutron capture therapy (BNCT), glioblastoma, 3D cell models, in vitro BNCT effect, *C. elegans*

## Abstract

**Simple Summary:**

Glioblastoma multiforme (GBM) is one of the most common malignant brain tumors. Although a variety of GBMs is initially susceptible to chemotherapy, the development of multi-drug resistance and recurrence is frequent. Therefore, there is an urgent need for more efficient treatment modalities for GBM. Boron neutron capture therapy (BNCT) is a cancer therapy based on the potential of ^10^B atoms to produce α particles that cross tissues in which the ^10^B accumulates without damaging the surrounding healthy tissues after irradiation with low energy thermal neutrons. The aim of our study was to assess that the sodium salt of cobaltabis(dicarbollide) and its di-iodinated derivative could be good candidates for dual anti-cancer treatment (chemotherapy + BNCT). Our results strongly suggest that these small molecules, in particular [8,8′-I_2_-*o*-COSAN]^−^, are serious candidates to be taken into account for BNCT now that the accelerator-based neutron source facilities are more accessible, providing an alternative treatment for resistant glioblastoma.

**Abstract:**

Purpose: The aim of our study was to assess if the sodium salt of cobaltabis(dicarbollide) and its di-iodinated derivative (Na[*o*-COSAN] and Na[8,8′-I_2_-*o*-COSAN]) could be promising agents for dual anti-cancer treatment (chemotherapy + BNCT) for GBM. Methods: The biological activities of the small molecules were evaluated in vitro with glioblastoma cells lines U87 and T98G in 2D and 3D cell models and in vivo in the small model animal *Caenorhabditis elegans* (*C. elegans*) at the L4-stage and using the eggs. Results: Our studies indicated that only spheroids from the U87 cell line have impaired growth after treatment with both compounds, suggesting an increased resistance from T98G spheroids, contrary to what was observed in the monolayer culture, which highlights the need to employ 3D models for future GBM studies. In vitro tests in U87 and T98G cells conclude that the amount of ^10^B inside the cells is enough for BNCT irradiation. BNCT becomes more effective on T98G after their incubation with Na[8,8′-I_2_-*o*-COSAN], whereas no apparent cell-killing effect was observed for untreated cells. Conclusions: These small molecules, particularly [8,8′-I_2_-*o*-COSAN]^−^, are serious candidates for BNCT now that the facilities of accelerator-based neutron sources are more accessible, providing an alternative treatment for resistant glioblastoma.

## 1. Introduction

Glioblastoma multiforme (GBM) is one of the most common malignant brain tumors among adults and the second most common cause of cancer-related deaths in children under the age of 15 [1]. It has a poor prognosis, and the life expectancy of patients is approximately one year [2]. Even with treatment, almost all GBMs develop resistance to therapy and recurrence [3,4,5]. Therefore, there is an urgent need for more efficient treatment modalities for GBM. In that context, boron neutron capture therapy (BNCT) has emerged as a therapeutic strategy that aims at selectively destroying tumors by targeting tumor cells with ^10^B-containing compounds, followed by deposition of high doses of radiation on those cells through a thermal neutron capture reaction to yield high linear energy transfer (high-LET) α particles and recoiling ^7^Li nuclei [6]. Since the path’s length of the α-particle is approximately 9–10 μm, similar to a single cell diameter, it is possible to selectively irradiate a tumor cell with a large dose of radiation. In theory, the labeled cells will die, whereas the unlabeled ones will remain undamaged even if located in close vicinity to the target cells.

**Figure 1:**
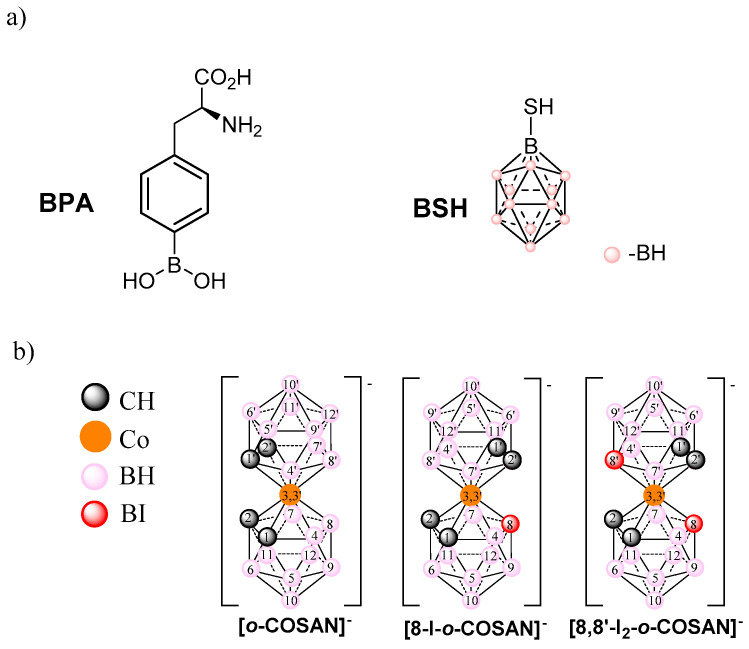
Schematic representation: (**a**) BPA and BSH, clinically used in the treatment of cancer with BNCT; (**b**) the sodium salts of cobaltabis(dicarbollide) clusters [3,3′-Co(1,2-C_2_B_9_H_11_)_2_]^−^, [3,3′-Co(8-I-1,2-C_2_B_9_H_10_)(1′,2′-C_2_B_9_H_11_)]^−^, and [3,3′-Co(8-I-1,2-C_2_B_9_H_11_)_2_]^−^, abbreviated as [*o*-COSAN]^−^, [8-I-*o*-COSAN]^−^, and [8,8′-I_2_-*o*-COSAN]^−^, respectively.

The common challenges in BNCT include the development of novel and improved boron agents, more accurate radiation dosimetry, and the neutron sources that currently depend on accelerators [7,8,9,10]. Two compounds *p-*borono-l-phenylalanine (BPA) and disodium mercapto-*closo*-undecahydrododecaborate (BSH) are clinically used for BNCT (Figure 1) [11,12,13]. BPA is selectively taken up into the tumor cells, while BSH has slightly low tumor selectivity [14]. However, BSH and its derivatives are of increasing interest as they carry a large load of ^10^B atoms to the tumorous cells (12 times more B per BSH-molecule than BPA) [15,16,17]. The first boron drug Steboronine ^®^ to be employed by BNCT was developed by Stella Pharma Corporation recently [18]. BNCT not only provides an alternative option to the oncologists for the GBM treatment but can also be advantageous for treating locally unresectable recurrent or advanced head and neck cancer.

In the field of medicinal chemistry, in particular the development of novel boron-based compounds, efforts have been made to improve selectivity as well as stability, solubility, and safety for a wide diversity of prospective BNCT drugs [19]. We have developed novel compounds based on the anionic cobaltabis(dicarbollide) [3,3′-Co(1,2-C_2_B_9_H_11_)_2_]^−^, [*o-*COSAN]^−^, which could be used as novel BNCT’s drugs. Indeed, [*o-*COSAN]^−^, in which Co^3+^ ion is sandwiched between two [7,8-C_2_B_9_H_11_]^2−^ ligands (Figure 1), rank among the most chemically and biologically stable small molecular compounds known [20] with a global negative charge, spread all over the molecule [21]. The anionic [*o-*COSAN]^−^ cluster, which incorporates the redox properties of the metal [22], displays low toxicity in vitro and in vivo due to their inertness to biochemical reactions [23,24,25,26,27,28]. Furthermore, the anionic cluster owing to its 3D aromatic character [29], produces hydrogen and dihydrogen bonds (C_c_-H···O and C_c_-H···H-B or N-H···H-B, respectively; C_c_ represents the C cluster atoms) [30,31] that have been proven to participate in its water solubility [32], self-assembly [30,31,33], and in micelles and vesicles formation [34,35,36,37]. Previously, some of the authors demonstrated that Na[*o*-COSAN] has potential for therapeutic modalities such as BNCT due to its high boron content, low toxicity, high uptake by relevant cancer cells [38], accumulation in vitro within living cells’ nucleus, and its capacity to strongly interact with DNA and proteins [27]. Recently, the amphiphilic inorganic anionic [*o*-COSAN]^−^ cluster has been proposed to stabilize oil-in-water nano-emulsions of poorly water-soluble drugs [39]. Additionally, the cluster can be modified at different vertexes by halogenation [40,41,42,43,44,45,46], which are responsible for additional physicochemical properties [20]. Furthermore, the in vivo imaging using [*o*-COSAN]^−^ as a molecular imaging platform was achieved by radiolabeling the monoiodinated [3,3′-Co(8-I-1,2-C_2_B_9_H_10_) (1′,2′-C_2_B_9_H_11_)]^−^ cluster, abbreviated as [8-I-*o*-COSAN]^−^ (Figure 1). Radiolabeling produced by either ^125^I (gamma emitter) or ^124^I (positron emitter), which enabled the determination of the biodistribution pattern by using dissection/gamma counting and positron emission tomography in combination with X-ray computed tomography (PET-CT), respectively [26]. Focusing on GBM, the use of boron delivery agents in most patients is usually preceded by positron emission tomography (PET) imaging. In that aspect, the possibility of using the long-lived positron emitter ^124^I (t_1/2_ = 4.2 d) to prepare the radioiodinated Na[8,8′-^124^I_2_-*o*-COSAN] also constitutes an attractive perspective, increasing the relevance of these compounds for treatment planning, in addition to improving the therapeutic results.

The small anionic molecule [*o-*COSAN]^−^ and its di-iodinated derivative [3,3′-Co(8-I-1,2-C_2_B_9_H_10_)_2_]^−^, abbreviated as Na[8,8′-I_2_-*o*-COSAN], possess the ability to readily cross biological membranes without disrupting membrane’s integrity [47,48] as well as artificial planar membranes even after being negatively charged [48] and accumulates inside the living cells with no appreciable cytotoxic effect [38]. However, a cytostatic effect was observed over long-term culture, and the cells recovered upon its removal. Multifunctional anti-tumor agents (either chemo- or radio-active) overcome resistance and act in two or more ways offering greater therapeutic benefits over single-mechanism entities.

In this study, the potential small anionic molecules [*o*-COSAN]^−^ and [8,8′-I_2_-*o*-COSAN]^−^ that offer dual mechanism, chemo-action and neutron radiotherapy, which could provide significant clinical benefits for the treatment of glioblastoma using BNCT, was assessed in vitro and in vivo using methodologies mimicking more realistic environments. We evaluate the biological activities of these small anionic molecules in vitro with U87 and T98G glioblastoma in 2D and 3D cell models and in vivo in the small model animal *Caenorhabditis elegans* (*C. elegans*). Additionally, we tested these small molecules in vivo using the small model animal *C. elegans* at the young adult L4-stage and using the eggs, which allowed us to assess the crossing ability in vivo of the [*o*-COSAN]^−^ and [8,8′-I_2_-*o*-COSAN]^−^. To sum up, [*o*-COSAN]^−^ and [8,8′-I_2_-*o*-COSAN]^−^ can offer the possibility of acting as chemotherapeutics and also be promising for BNCT, which would provide significant clinical benefits for the treatment of glioblastoma.

## 2. Materials and Methods

### 2.1. Materials

Cs[*o-*COSAN] was synthesized from 1,2-*closo*-C_2_B_10_H_12_ from Katchem Spol.sr.o (Kralupy nad Vltavou, Czech Republic) as reported in the literature [49]. [NMe_4_] [8,8′-I_2_-*o*-COSAN] was synthesized from Cs[*o-*COSAN] as described [50]. The sodium salts of [*o-*COSAN]^--^ and [8,8′-I_2_-*o*-COSAN]^−^ species were obtained by means of cationic exchange resin of the corresponding cesium and tetramethyl ammonium salts, respectively [51].

### 2.2. Instrumentation and Measurements

The FTIR experiments were performed on a Shimadzu FTIR-8300 spectrophotometer. The ^11^B, ^11^B{^1^H} NMR (128.37 MHz) and NMR (400 MHz) spectra were obtained using a Bruker ARX 400 instrument equipped with appropriate decoupling accessories with deuterated water as the solvent at 22 °C. The chemical shifts of the boron and proton spectra were referenced to the external BF_3_·OEt_2_ and internal SiMe_4_, respectively, and are reported as parts per million downfield from the reference. The UV-vis experiments were done on Jasco V-780 spectrophotometer, using 1 cm quartz cuvettes for metallabis(dicarbollides) (0.08 mM in water) and 1 mm cuvettes for *C. elegans*’ embryos extraction supernatants. To measure the hydrodynamic diameter of the samples, dynamic light scattering (DLS) studies were performed using Zetasizer nano ZS (Malvern Instruments Ltd., Cambridge, UK) equipped with a He-Ne 633 nm laser using disposable cuvettes. These studies were done using 1 mL of sample’s dispersion in water, which was filtered before measuring using a syringe filter (PTFE, 0.2 μm pore diameter). For each sample, the measurements were run in triplicate at ambient temperature with multiple sub-runs. Transmission electron microscopy (TEM) studies were carried out on JEOL JEM 1210 at 120 kV. Vitrified specimens were prepared by placing 3 µL of 1 mM suspension of Na[*o*-COSAN] on a 400-mesh copper grid with holey carbon support. Each of the samples was blotted to a thin film and immediately plunged into liquid ethane in the Leica CPC cryo-workstation. The grids were observed on a JEOL 2011 transmission electron microscope operating at 200 kV equipped with a Gatan cryoholder, and the samples were maintained at −177 °C during imaging. Electron micrographs were obtained with the Digital Micrograph software package (Lousiana State University, Baton Rouge, LA, USA) under low electron dose conditions, in order to minimize the electron beam radiation. The images were recorded on a Gatan 794 MSC 600HP cooled charge-coupled device (CCD) camera. Scanning electron microscope (SEM) experiments were performed using Quanta FEI 200 FEG-ESEM operating at 15 kV and low vacuum (60 Pa). The microscope was equipped with an Oxford Inca Energy Dispersive X-ray (EDX) system for chemical analysis, where both qualitative and quantitative analysis could be performed by using the software Genesis Spectrum version 5.21 (EDAX INC., Mahwah, NJ, USA).

### 2.3. Cell Lines

The human glioblastoma cell lines U87 and T98G, acquired from ATCC, were cultivated in EMEM medium (Lonza, Basel, Switzerland) containing the following supplements: 10% FBS (Lonza, Basel, Switzerland), 1 mM sodium pyruvate, 2 mM glutamine, non-essential amino acids, and 1% antibiotics. Cells were routinely grown in a humidified atmosphere with 5% CO_2_ at 37 °C.

### 2.4. Metallabis (Dicarbollides) Solutions Preparation

Fresh stock solutions (1 mM) of the sodium salts, Na[*o*-COSAN] and Na[8,8′-I_2_-*o*-COSAN], and serial dilutions from the stock were prepared in a complete growth medium.

### 2.5. Cell Viability of Monolayer Cultures

Cell viability was assessed using the MTT assay following a method similar to the one previously described [27]. Briefly, U87 and T98G cells (0.8 − 5 × 10^4^ cells/well) were seeded in 96-well plates and left to adhere. After 24 h, cells were incubated with increasing concentrations of [*o*-COSAN]^−^ or [8,8′-I_2_-*o*-COSAN]^−^ from 1 µM to 1000 µM for 6, 24, 48, and 72 h. After incubation, the medium was removed, and each well was incubated with an MTT solution (5 mg/mL in medium) for 45 min at 37 °C. Then, the medium was discarded, and the formazan crystals were solubilized in 150 μL DMSO. Absorbance was measured at 570 nm using an iMark microplate reader (Biorad, Hercules, CA, USA) or a Varioskan ^TM^ LUX microplate reader (Thermo Scientific, Waltham, MA, USA).

### 2.6. 3-D Spheroid Cultures

U87 and T98G spheroids were prepared in Nunclon ™ Sphera ™ ultra-low attachment 96U-well plates as described [52]. Briefly, 1000 and 4000 cells of U87 and T98G, respectively, were plated per well before the plates were centrifuged and incubated at 37 °C in a humidified atmosphere of 5% CO_2_. Spheroid growth was assessed every day by optical microscopy, and images were analyzed using the free software SpheroidSizer.zip (http://pleiad.rwjms.rutgers.edu/CBII/downloads/SpheroidSizer.zip (accessed on 15 September 2021)). On day 3, when the spheroids had reached diameters in the range of 350–400 μm, the spheroids’ viability was determined.

### 2.7. Cell Viability of Spheroid Cultures

The spheroids’ viability was determined as described [52]. Briefly, 100 μL of culture medium were removed from each well containing three-day-old U87 or T98G spheroids, and 100 μL of each compound was added, corresponding to concentrations of each compound and for each cell line that had been previously selected. Control spheroids were incubated with 100 μL of complete growth medium containing no compounds. After 72 h of incubation, viability was determined using the APH assay. Three independent assays were performed, using at least 5 spheroids per condition in each assay. In parallel, U87 and T98G cells in monolayer incubated in the same conditions were also tested in 96 well plates with the APH assay (three independent assays with 5 wells per condition).

### 2.8. In Vivo Studies in C. elegans

*C. elegans* Bristol strain N2 and *Escherichia coli OP50* were obtained from the Caenorhabditis Genetic Center. Nematodes were maintained using standard procedures [53], and exposure towards materials was performed following previous protocols [54].

Synchronized L4 were treated 24 h with Na[*o-*COSAN] and Na[8,8′-I_2_-*o-*COSAN] at concentrations ranging from 0 to 50 µM in M9 buffer supplemented with a 4% of dead OP50 in a final volume of 100 μL. After exposure, worms’ survival was computed by tapping the plate and counting the moving alive worms [54]. Each well contained 15 ± 3 L4 worms (number of total worms (*n*) = 450, number of independent experiments (N) = 3. LD_50_ was calculated using GraphPad Prism 9 and fitting the curve to a four-parameters dose-response curve.

**Scheme 1:**
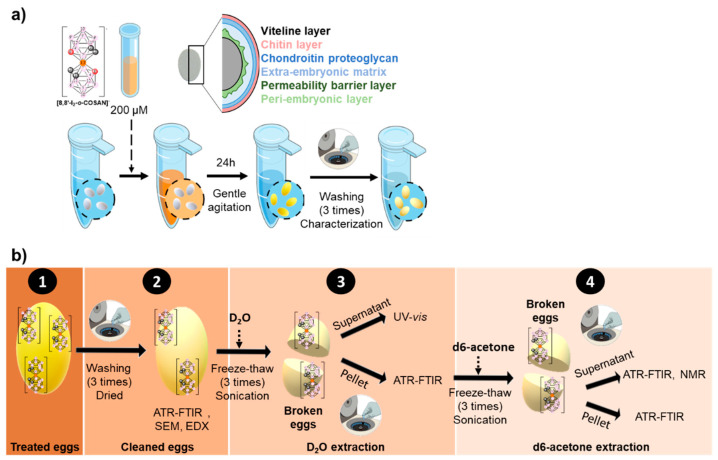
(**a**) Schematic representation of the C. elegans embryos treatment with Na[8,8′-I_2_-*o*-COSAN]. (**b**) Designed procedure for the study of formed new hybrids eggs/[8,8′-I_2_-*o*-COSAN]^−^.

The interaction with *C. elegans’* embryos (Scheme 1a) was evaluated using embryos freshly harvested following standard synchronization protocols with alkaline treatment (5N NaOH and household bleach (1:2) is added to a final concentration (1:10)) [54]. Approximately 4.5 × 10^3^ eggs/mL were exposed to 200 µM concentration of each compound in M9 buffer for 24 h at RT under gentle agitation. After the exposure (Scheme 1b), eggs were washed 3 times with MQ water to remove the compounds’ excess; isolated by centrifugation (5000 rpm, 1 min), imaged using inverted optical microscope Olympus IX53 and then, dried (60 °C, 17 h). These dried pellets were studied by ATR-IR (FTIR-4700 JASCO, Madrid, Spain) in the range 400–4000 cm^−1^ (64 scans) and by SEM/EDX (FEI Quanta 650FEG ESEM) under low vacuum condition, with an acceleration voltage of 15 kV and electron beam spot of 3.5 and a working distance of 9.9 mm. Four drops of 3 µL of each sample were dried (60 °C, 17 h) on top of an aluminum SEM holder with adhesive carbon tape. An energy-disperse X-ray (EDX) scan was used to determine the elements present in the sample.

To break the egg’s shell and extract the water-soluble compounds, dried pellets (named as eggs-control, eggs-[*o*-COSAN]^−^, and eggs-[8,8′-I_2_-*o*-COSAN]^−^) were suspended firstly in deuterated water (D_2_O) and then frozen with N_2_ and thawed at 37 °C three times, and sonicated at 80 °C for 1 h. This D_2_O solution was analyzed by UV-vis spectroscopy while the remaining solid was dried and analyzed by ATR-IR. Then, deuterated acetone was added to the dried pellet; this d_6_-acetone solution was analyzed by ATR-IR and RMN (^1^H{^11^B} and ^11^B{^1^H}) spectroscopies. The remaining pellet was also analyzed by IR to assess the presence of the compound in cell debris (Scheme 1b).

### 2.9. Uptake Experiments

For the in vitro uptake experiments, U87 and T98G cells were seeded at a density of 5 × 10^5^ in 6 cm of diameter dishes and then incubated with increasing concentration of [*o*-COSAN]^−^ or [8,8′-I_2_-*o*-COSAN]^−^ (1 µM, 2 µM, 3 µM, 5 µM, 10 µM, and 20 µM) for 24 h in condition of normoxia at 37 °C, 5% CO_2_. After the incubation, the medium was discarded, and the cells were rinsed three times with 3 mL ice-cold PBS and detached with 0.05% trypsin and 0.02% EDTA. Finally, cell pellets were transferred to falcon tubes and sonicated for 30 s at 30% of power in ice. The protein content of each cell sample lysate was determined by Bradford assay (Biorad, CA, USA) using bovine serum albumin as a standard. Cell lysates were then mineralized in 70% nitric acid, and boron content was determined by inductively coupled plasma mass spectrometry (ICP-MS) technique.

### 2.10. Inductively Coupled Plasma Mass Spectrometry (ICP-MS)

The B content from collected cell samples was measured using ICP-MS (Element-2; Thermo-Finnigan, Rodano (MI), Italy). Sample preparation was carried out using a high-performance Microwave Digestion System (ETHOS UP Milestone, Bergamo, Italy) after the addition of concentrated HNO_3_ (70%) to cell lysates (1:1) in a final volume of 0.4 mL. Boron content was then normalized to the protein concentration of each sample, which was proportional to the number of cells by means of a calibration curve (mg protein/number of cells). Considering that 1 g of tissue contains 1 × 10^9^ cells, the amounts of boron expressed as μg/g tissue were thus determined. In order to check for variations in the systematic bias, both natural boron abundant and ^10^B enriched standard solutions were used during sample runs. Four boron absorption standard solutions (Sigma-Aldrich, St. Louis, MO, USA), with a concentration in the range 0.1–0.004 μg/mL, were used to obtain calibration curves.

### 2.11. Neutron Irradiation Experiments

U87 and T98G cells were seeded in 75 flasks (4 flasks for each cell line) at a density of 1.5 × 10^6^ cells. After 24 h from seeding, 2 flasks of each cell line were incubated in the presence of 20 μM of [*o*-COSAN]^−^ or [8,8′-I_2_-*o*-COSAN]^−^ for 24 h. Cells were then washed with PBS, and a new medium was added. The thermal column of the TRIGA Mark II reactor at the University of Pavia (Italy) was used for neutron irradiation of cells. Two flasks containing treated and one flask containing non-treated control U87 and T98G cells have been used for this experiment. The irradiation power time and power were 15′ and 30 kW, respectively. At the end of the irradiation, the medium was discarded, replaced with a fresh one, and all the flasks were placed at 37 °C in a humidified atmosphere of 5% CO_2_.

Twenty-four hours after irradiation, cells were detached with 0.05% trypsin and 0.02% EDTA, and cell viability was evaluated using the trypan blue exclusion test and reported as the percentage of cells observed in treated a/o irradiated samples with respect to that observed in control cells.

### 2.12. Statistical Analysis

The data presented in the paper are shown as mean values ± standard error of mean or mean values ± standard deviation, as indicated, and, unless otherwise stated, at least 3 biological replicates were used. Statistical analysis was performed using the GraphPad Prism 6 software (GraphPad Software, San Diego, CA, USA) in order to assess if there was an effect of each treatment compared with the respective control samples. For that, a one-way ANOVA followed by Dunnett’s test was performed with a threshold of *p* ≤ 0.05.

## 3. Results and Discussion

### 3.1. Physical-Chemical Properties of the Sodium Salts of [o-COSAN]^−^ and [8,8′-I_2_-o-COSAN]^−^

#### 3.1.1. Solubility and Lipophilicity of Na[o-COSAN] and Na[8,8′-I_2_-o-COSAN]

For biological and clinical applications, it is crucial to evaluate the compounds’ behavior in an aqueous solution. Solubility and lipophilicity are basic but key physicochemical parameters that can dictate the success or failure of drug discovery and development. The concentration (mol/L) of a saturated aqueous solution of a compound (S) is named aqueous solubility, and it is an important parameter to consider because it affects its bioavailability [55,56,57]. This aqueous solubility of S is almost exclusively dependent on the intermolecular interactions between molecules, molecule–water, and water–water. Thus, several factors such as size and shape, the polarity and hydrophobicity of the molecule, in addition to the ability to participate in intra- and intermolecular hydrogen or dihydrogen bonding, affect the solubility of S.

Furthermore, a key physicochemical parameter linking solubility, membrane permeability, and hence, drug absorption and distribution is its lipophilicity (P), usually expressed by octanol/water partition or distribution coefficients (logP/logD). Lipophilicity is a major factor influencing passive brain transfer across the BBB in either direction. Table 1 summarized the parameters of both anionic cobaltabis(dicarbollides), [*o*-COSAN]^−^ and [8,8′-I_2_-*o*-COSAN]^−^, related to solubility and lipophilicity.

Typically, for a radiotracer to be considered as a potential efficient molecular imaging probe in the living human brain with positron emission tomography (PET), its partition coefficients (logP) should range between 2.0 and 3.5 [58]. The logP of the di-iodinated Na[8,8′-I_2_-*o*-COSAN] complex is within this range—2.18 [38].

#### 3.1.2. Dynamic Light Scattering and Transmission Electron Microscopy

It has been reported that [*o-*COSAN]^−^ and its halogenated anionic derivatives [3,3′-Co(1,2-C_2_B_9_H_8_X_3_)_2_]^−^ (X = H, Cl, Br) behave likewise to a surfactant in aqueous solutions [59] and that [*o*-COSAN]^−^ organizes in spherical aggregates with a radium of around 100 nm in a fairly monodisperse way, which depends on the concentration and aging of the solutions [60]. As the ^1^H{^11^B} NMR spectra of both [*o*-COSAN]^−^ and [8,8′-I_2_-*o*-COSAN]^−^ cobaltabis(dicarbollides), provided information on the aggregates formation [51], we have performed the dynamic light scattering (DLS) of Na[8,8′-I_2_-*o*-COSAN] in aqueous solutions at different concentrations to study the effect of the iodine atoms and to compare its aggregates formation with the reported parent Na[*o*-COSAN].

**Figure 2:**
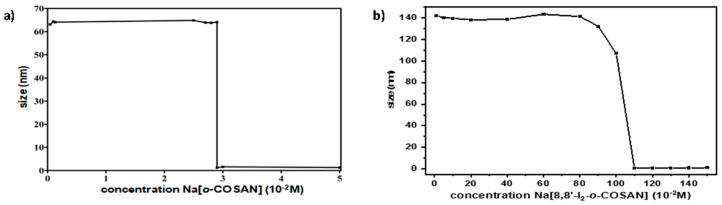
DLS studies of (**a**) Na[*o-*COSAN]: concentration range 1–50 mM; (**b**) Na[8,8′-I_2_-*o*-COSAN]: concentration range 5–150 mM in aqueous solution.

Figure 2 displays the DLS measurements, which reveal the presence of a large number of aggregates in Na[8,8′-I_2_-*o*-COSAN]. In both clusters, a decrease in the hydrodynamic diameter of the aggregates was observed by DLS experiments when the concentration was increased, which can be attributed to a Coulomb explosion [61,62,63,64] of the closely packed monolayer aggregates into small micelles. The change in size for Na[8,8′-I_2_-*o*-COSAN] occurred at a concentration of 110 mM, below which the size of the aggregates was 107 nm and above 1.11 nm, while for Na[*o-*COSAN], the change in size occurred at a concentration of 30 mM, below which vesicles of size around 63 nm were observed.

**Figure 3:**
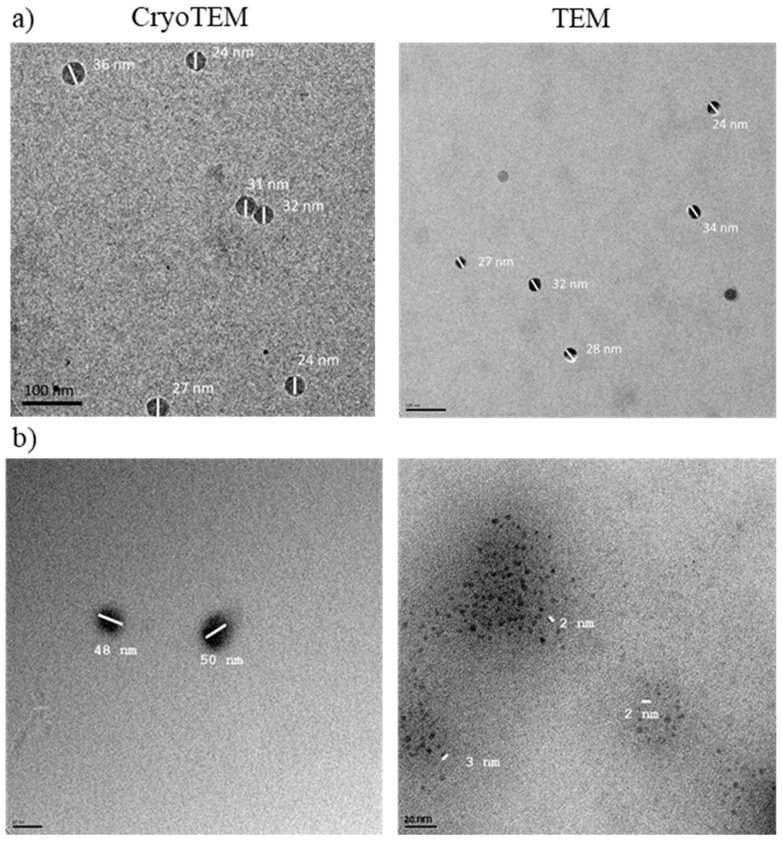
(**a**) CryoTEM and TEM images of an aqueous solution 1 mM of Na[*o*-COSAN]; bar length is 100 nm. (**b**) TEM image of aqueous solutions 60 mM (left) and 120 mM (right) of Na[8,8′-I_2_-*o*-COSAN]; bar length is 20 nm.

In order to visualize the aggregates of Na[8,8′-I_2_-*o*-COSAN], TEM images were taken (Figure 3b). In the case of the parent Na[*o*-COSAN] cluster, CryoTEM images of an aqueous solution 1 mM were also obtained for comparison (Figure 3a).

#### 3.1.3. Infrared and NMR Studies

FTIR and NMR spectroscopies are essential analytical tools for structure elucidation and chemical determination. Recently [65], it has been reported by using synchrotron radiation-based Fourier transform infrared (SR-FTIR) that Na[*o*-COSAN] strongly interacts with proteins modifying their secondary structure after its uptake by two different glioma initiating cells (mesenchymal and proneural). This result has been possible because icosahedral Boron clusters (boranes, carboranes, and metallacarboranes) display strong and characteristic ν(B-H) frequencies in the infrared spectral range of 2.600–2.500 cm^−1^ where no other frequencies of compounds appear [66]. The interaction between Na[*o*-COSAN] and biomolecules can also be observed by ^11^B{^1^H}NMR spectroscopy [27].

**Figure 4:**
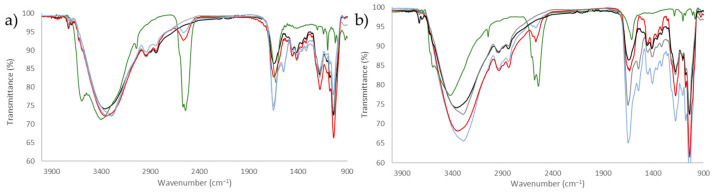
(**a**) IR spectra of 2 mM of Na[*o*-COSAN] in aqueous solution (green), in DMEM culture medium solution (red), in DMEM + 10% FBS solution (blue), DMEM culture medium solution (black), and DMEM + 10% FBS solution (grey). (**b**) IR spectra of 2 mM of Na[8,8′-I_2_-*o*-COSAN] in aqueous solution (green), 2 mM of Na[*o*-COSAN] in DMEM culture medium solution (red), 2 mM of Na[8,8′-I_2_-*o*-COSAN] in DMEM + 10% FBS solution (blue), DMEM culture medium solution (black), and DMEM + 10% FBS solution (grey).

**Figure 5:**
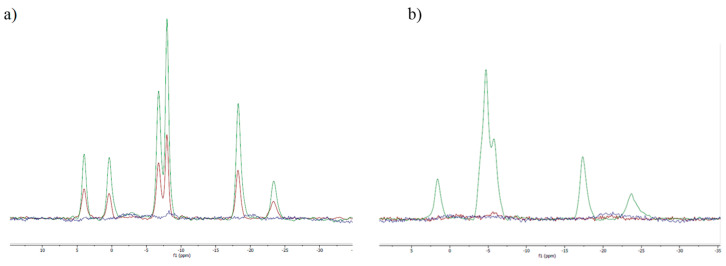
(**a**) ^11^B{^1^H}-NMR spectra of 2 mM of Na[*o-*COSAN] in aqueous solution (green), in DMEM culture medium solution (red), in DMEM + 10% FBS solution (blue). (**b**) ^11^B{^1^H}-NMR spectra of 2 mM of Na[8,8′-I_2_-*o-*COSAN] in aqueous solution (green), in DMEM culture medium solution (red), in DMEM + 10% FBS solution (blue).

Both spectroscopies, FTIR and NMR, have been run to provide information on the possible interaction between Na[*o-*COSAN] and Na[8,8′-I_2_-*o*-COSAN], DMEM (without phenol red) culture medium, and DMEM + 10% FBS. FTIR and ^11^B{^1^H}NMR spectra of 2 mM solutions of Na[*o*-COSAN] and Na[8,8′-I_2_-*o*-COSAN] in H_2_O, DMEM, and DMEM + 10% FBS were carried out (Figure 4 and Figure 5).

Figure 4 displays that the characteristic ν(B-H) frequency of the Na[*o*-COSAN] decreases in DMEM culture medium (Figure 4, red) concerning in aqueous solution but the ν(B-H) band intensity decreases more if the culture medium contains FBS (Figure 4 blue); to emphasize that this frequency is not present in the DMEM culture medium nor in the culture medium DMEM + 10% FBS (Figure 4, black and grey, respectively).

Figure 5a clearly shows that the ^11^B{^1^H} NMR resonances corresponding to the Na[*o*-COSAN] solution in DMEM (without phenol red) culture medium (Figure 5a, in red) diminish with respect to the signals of Na[*o*-COSAN] in aqueous solution (Figure 5a, in green) and fully disappear in the ^11^B{^1^H} NMR spectrum of Na[*o*-COSAN] in DMEM culture containing 10% FBS protein (Figure 5a, in blue). The latter result is analogous to the previous Na[*o*-COSAN] study performed in DMEM medium in the presence of 10% FBS [27], which supports that Na[*o*-COSAN] forms protein aggregates. Similar trends were observed for Na[8,8′-I_2_-*o*-COSAN] (Figure 5b).

### 3.2. Cell Viability Assays

The in vitro assessment of the inhibitory capacity of [*o*-COSAN]^−^ and [8,8′-I_2_-*o*-COSAN]^−^ was performed by evaluating cell viability by means of the MTT assay. The experiments were carried out by adding increasing amounts of [*o*-COSAN]^−^ or [8,8′-I_2_-*o*-COSAN]^−^ to the incubation medium containing the cells (U87 and T98G, respectively) for 24 h in normoxic condition (at 37 °C and 5% CO_2_ in a cell incubator). Table 2 reports that the percentage of viable cells steadily decreases upon increasing the concentration of both [*o*-COSAN]^−^ and [8,8′-I_2_-*o*-COSAN]^−^. For both cell lines, [8,8′-I_2_-*o*-COSAN]^−^ was found to be more toxic than [*o*-COSAN]^−^. This has already been reported in previous papers for other cell lines [39] and is presumably due to the greater ability of [8,8′-I_2_-*o*-COSAN]^−^ in crossing biological membranes compared to [*o*-COSAN]^−^ [47,48]. In Table 2, the IC_50_ values of the two different compounds for both cell lines determined for different incubation periods are reported. [*o*-COSAN]^−^ is not cytotoxic up to 48 h of incubation in the U87 cells. However, at 48 h incubation, this compound is more active in the T98G cells. At this incubation time, the corresponding iodinated compound seemed to be even more active in both cell lines. Data from Table 2 reveal that for incubation times longer than 24 h, the T98G cells were shown to be more sensitive to both [*o*-COSAN]^−^ and [8,8′-I_2_-*o*-COSAN]^−^ in comparison to the U87 cells.

Results on the cytotoxic behavior of these compounds were surprising, taking into consideration other reports in the literature on the characteristics of these glioblastoma (GMB) cells. In fact, in contrast with U87 cells, the T98G cells are reported as chemoresistant (Temozolomide/TMZ) GMB cells [67]. Therefore, our results suggest that these compounds, in particular the [8,8′-I_2_-*o*-COSAN]^−^ are worth further investigating either alone or in combination with other conventional chemotherapeutics, in a larger panel of chemo-resistant GBM cell lines.

### 3.3. Effect of Cobaltallabis (Dicarbollides) in GBM Spheroids’ Viability

The spheroids were exposed to the two metallabis(dicarbollides) on the third day of culture in three different dosages. The concentrations of each compound were chosen based on the IC_50_ values previously determined for the monolayer culture of this cell line at 72 h, using the MTT assay (Table 3), and then using a lower and a higher concentration than those IC_50_ values (0.5 × IC_50_ and 1.25 − 5 × IC_50_). After 72 h of exposure to the compounds (6th day of spheroid culture), the viability of the U87 and T98G spheroids was assessed by the acid phosphatase (APH) assay. Physical characterization of the spheroids upon incubation with the compounds, which included the measurement of the diameter, area, and circularity, was also performed daily, from day 3 to day 6 of culture.

**Figure 6:**
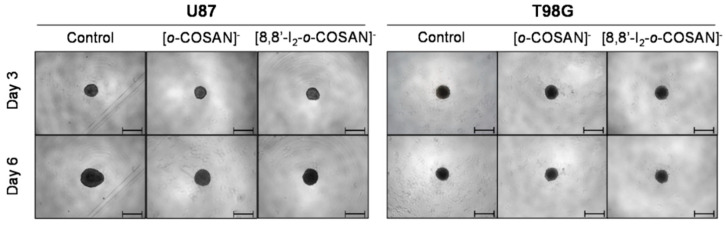
Representative images of the U87 and T98G spheroids before (day 3 of culture) and after 72 h (day 6 of culture) of exposure to the two compounds at their IC50 concentration. Controls consist of spheroids incubated only with medium. Scale bars correspond to 500 μm.

Illustrative microscope images of the spheroids exposed to the IC_50_ concentrations of the compounds tested are shown in Figure 6, while the images for the other concentrations tested (lower than IC_50_ and higher than IC_50_) are shown in Figures S1 and S2.

**Figure 7:**
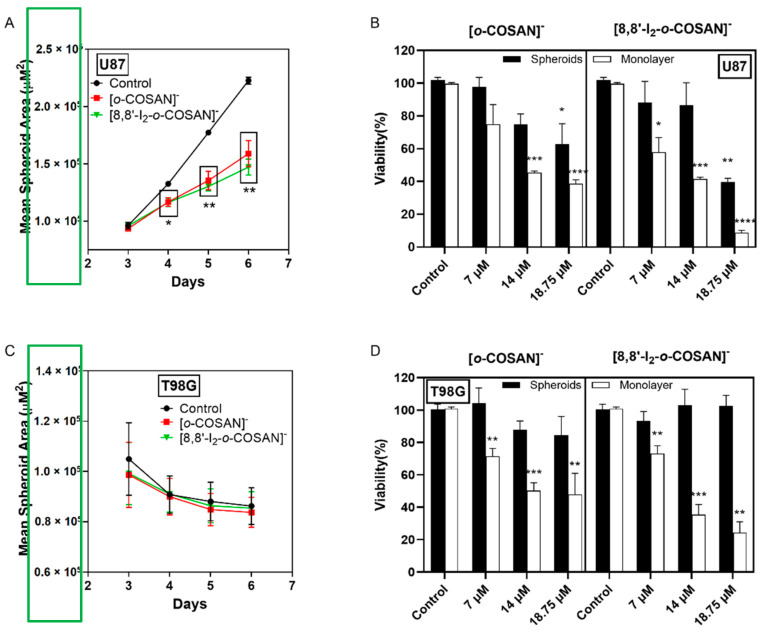
Effects of incubation of [*o*-COSAN]^−^ and [8,8′-I_2_-*o*-COSAN]^−^ on U87 (top panels) and T98G (bottom panels) spheroids. (**A**) U87 and (**C**) T98G spheroids growth after incubation with IC_50_ concentrations of [*o*-COSAN]^−^ and [8,8′-I_2_-*o*-COSAN]^−^, represented by the mean spheroids area (in µM^2^) as a function of the number of days in culture, and (**B**) and (**D**) cellular viability (%) at 72 h, assessed by the APH assay, in parallel to monolayer cultured cells. Controls consist of spheroids or monolayer cultured cells incubated only with medium. Data are presented as the average ± SEM of 3 independent assays. Statistical significance was calculated using one-way ANOVA, followed by Dunnett’s test comparing treated spheroids/cells with control spheroids/cells (* *p* ≤ 0.05, ** *p* ≤ 0.01, *** *p* ≤ 0.001, and **** *p* ≤ 0.0001).

In a qualitative analysis, the administration of the compounds did not seem to significantly affect the shape and integrity of the spheroids. Quantitatively, concerning the growth and size of the spheroids, both compounds affected the growth of the U87 spheroids, with a statistically significant decrease being observed in the spheroids’ area at day 3 of treatment for all the three concentrations tested: the IC_50_ (Figure 7A), above IC_50_ (Figure S1), and even below IC_50_ (Figure S2) concentrations. In contrast, for the T98G cell line, no changes were detected in the growth of the spheroids for any of the concentrations tested (Figure 7C, Figures S1 and S2).

Overall, the circularity of the spheroids was not severely affected by the administration of the compounds, maintaining, in most cases, a regular shape, which is reflected by circularity values very close to 1 (Figure S3).

The viability of the spheroids after incubation with the metallabis(dicarbollides) for 72 h was determined using the APH assay and was performed in parallel also for monolayer-cultured cells. The viability results of the U87 and T98G spheroids for the compounds with all the concentrations tested are represented in Figure 7B,D, respectively. In general, these results reflect the growth behavior observed for the spheroids. There was an obvious decrease in the viability of the spheroids from U87 treated with both compounds. However, using the concentration corresponding to the IC_50_ previously determined in monolayer-cultured cells by the MTT assay, no compound led to a statistically significant decrease in the spheroids’ viability from both cell lines (Figure 7B,D). When using a higher concentration (Figure 7B), only spheroids from the U87 cell line were found to have reduced viability after incubation with both compounds, suggesting an increased resistance from T98G spheroids, contrarily to the observation in the monolayer culture.

The same assay was performed in parallel for monolayer-cultured cells, and a dose-dependent decrease in viability was observed in those conditions (see Figure 7B,D). When compared with 3D spheroids, both U87 and T98G cells grown as monolayer cultures were more sensitive to the compounds tested. In a way, the results reflected the disparity between 3D and monolayer cultures as 3D culture systems are frequently more refractory to anti-cancer treatments due to limited drug penetration and activation of several resistance mechanisms [68]. In fact, spheroid models can mimic the metabolic and proliferative gradients of in vivo tumors, with consequent changes in cellular phenotype and status, exhibiting multicellular chemoresistance.

### 3.4. In Vivo Tests with Caenorhabditis elegans (C. elegans)

**Figure 8:**
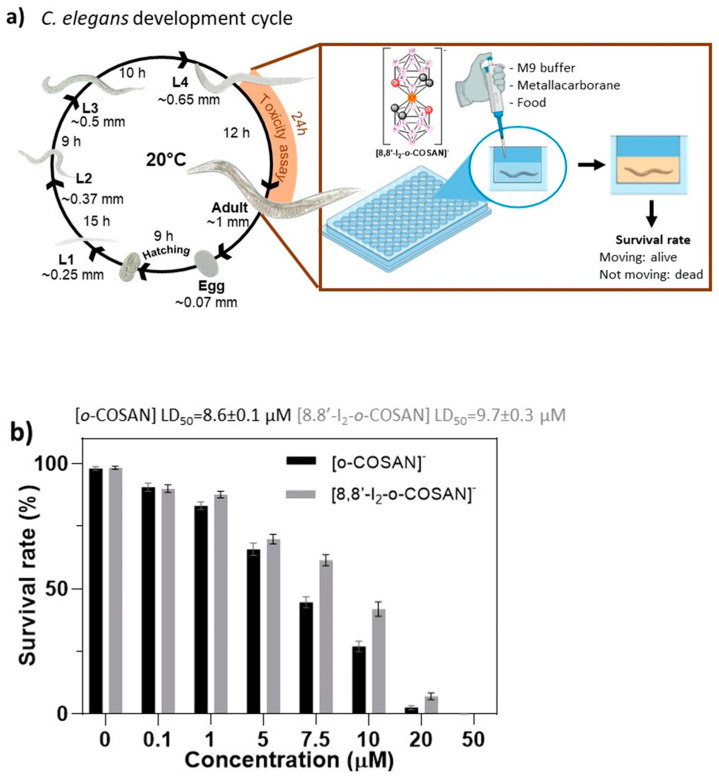
Toxicological evaluation of Na[*o*-COSAN] and Na[8,8′-I_2_-*o*-COSAN]. (**a**) Schematic representation of *C. elegans’* life cycle at 20 °C and exposure of the components from L4 stage to adult stage. (**b**) Survival rate of *C. elegans* after 24 h of exposure towards concentrations in a range 0–50 µM of Na[*o*-COSAN] (black) and Na[8,8′-I_2_-*o*-COSAN] (grey).

After performing in vitro testing of Na[*o-*COSAN] and Na[8,8′-I_2_-*o-*COSAN], we evaluated their toxicity in vivo using the small invertebrate *C. elegans*, which complement previous results and offer a physiologically relevant environment. *C. elegans* is a free-living nematode that, due to its simplicity, easiness of growth in large populations, and transparency, is used to assess the toxicity of drugs and materials [69,70] (Figure 8a, Scheme S1). Despite these advantages, we acknowledge that *C. elegans* is evolutionarily distant from humans, and it lacks some defined organs/tissues, including blood, a brain, and the ability to develop a tumor. However, the use of this worm in material science offers the possibility to screen different nanomaterials and drugs before going one step further, reducing time, cost, and experiments with complex animal models and therefore fulfilling the 3Rs rule (replacement, reduction, and refinement). With this information in hand, further evaluation in complex animal models could next be performed.

Synchronized L4 *C. elegans* were exposed to Na[*o-*COSAN] and Na[8,8′-I_2_-*o-*COSAN] at a concentration ranging from 0 to 50 µM for 24 h of incubation (Figure S4). Toxicity is easily screened by the survival of the worms (existence of movement) [69], facilitating the determination of the lethal dose 50% (LD_50_). LD_50_ for Na[*o-*COSAN] and Na[8,8′-I_2_-*o-*COSAN] were 8.6 ± 0.1 µM and 9.7 ± 0.3 µM, respectively; those values are close to the IC_50_ determined for T98G in vitro after 72 h (Figure 8b). The small anionic [*o*-COSAN]^−^ moieties, as described previously [71,72,73], could interact with the amine groups in the plethora of biomolecules of the organism and contribute to the toxicity.

The presence of both cobaltabis(dicarbollides) in the medium stop the development of worms at the L4 stage, whereas control worms could reach adulthood (Figures S4 and S5). Moreover, when worms were exposed to high concentrations of compounds (200 µM), a change towards yellowish color was observed, indicating accumulation in the body and the availability to cross *C. elegans* biological membranes in vivo.

Recently, the cell-cycle process of two glioma initiating cells (GICs: proneural GIC7 and mesenchymal PG88) treated with 200 µM Na[*o*-COSAN] 5 h was analyzed by flow cytometry [65]. When cells were recovered, 43 h after treatment, no differences were observed concerning control cells, neither GIC7 nor PG88. They might enter in the G2/M phase to complete cellular division, indicating that Na[*o*-COSAN] has a cytostatic effect in both GICs cells’ lines. Consequently, after the study with synchronized L4 *C. elegans*’ worms, we used *C. elegans*’ embryos to evaluate if the small cobaltabis(dicarbollide) anions would be able to interact with multilayer eggshell, crossing it and interfering with the embryonic development. *C. elegans*’ embryos are protected with a resistant and complex peri-embryonic shell of 300–400 nm thick [74], structured with different layers (vitelline, chitin, chondroitin, and extra-embryonic), whose chemical composition is polysaccharides, proteins, and lipids [74].

It was observed that *C. elegans*’ *e*mbryos did not develop to larvae stage after being treated with 200 µM in an M9 buffer solution of each compound (Na[*o-*COSAN] and Na[8,8′-I_2_-*o-*COSAN]) for 24 h at RT. This fact indicates that the compounds inhibit the development of embryos probably because of their interaction with the components of the protective shell, either at the peri-embryonic shell or at the cytoplasm.

Trying to elucidate on the interaction type along with on where the small anions ([*o-*COSAN]^−^ and [8,8′-I_2_-*o-*COSAN]^−^) locate, a thorough investigation study of the eggs was designed and performed (see Scheme 1). The extraction of different fractions at different steps and the analysis using different chemo-physical techniques such as optical and SEM microscopies, ATR-IR, UV-vis, EDX, and RMN spectroscopies allowed the identification of the COSAN moieties at the different structures.

**Figure 9:**
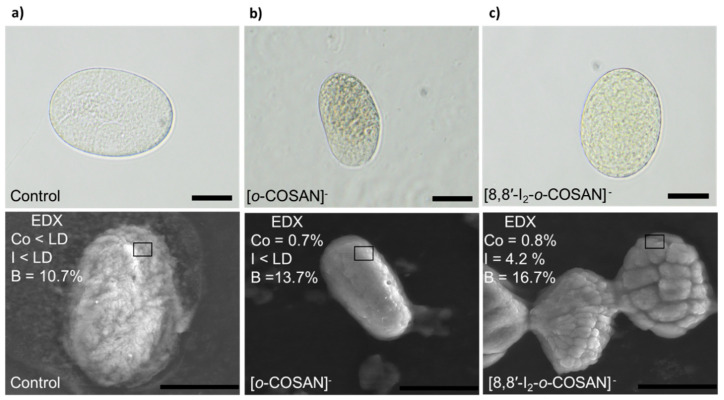
Optical and SEM images of the *C. elegans* embryos. (**a**) eggs-control; (**b**) after treatment with 200 μM of Na[*o-*COSAN] for 24 h at room temperature under gentle agitation; (**c**) after treatment with 200 μM of Na[8,8′-I_2_-*o-*COSAN] for 24 h at room temperature under gentle agitation. Bar size 20 μm.

Firstly, optical microscopy images of the treated eggs (after washing three times with M9) clearly indicated a change in the color (Figure 9a), which would agree with the presence of the small anions in the eggs. Then, the ATR-FTIR spectrum of the dry eggs’ samples (eggs-[*o*-COSAN]^−^ and eggs-[8,8′-I_2_-*o*-COSAN]^−^, respectively) (Figure S7) was run. The spectra displayed the characteristic B-H band around 2.550 cm^−1^ and a change in the fingerprint area (1233–400 cm^−1^ range) confirming the metallacarborane’s presence in the treated eggs and their interaction with eggs’ components [66]. EDX measurements of the dried eggs-[*o*-COSAN]^−^ and eggs-[8,8′-I_2_-*o*-COSAN]^−^ samples confirm the presence of cobalt in both samples as well as iodine in the eggs-[8,8′-I_2_-*o*-COSAN]^−^ (Figure 9 and Figures S8–S10).

The UV-vis spectra of the extracted aqueous solutions (Figure S11) also confirmed the presence of the anionic cluster (Figure S12). However, the residual pellets still displayed a clear yellow color (Figure S11), which may indicate that the metallacarboranes remained in both the treated embryos after aqueous extraction. The fact that the metallabis (dicarbollides) remained in the dried pellets was confirmed by ATR-IR spectra, which clearly displayed the band around 2550 cm^−1^ corresponding to the ν(B-H) (Figure S13). Taking into account that the sodium salts of the small anions, [*o-*COSAN]^−^ and [8,8′-I_2_-*o-*COSAN]^−^, are water-soluble (1509 and 210 mM, respectively) [32], the former unsuccessful extraction with D_2_O suggests that the small anionic molecules strongly interact with amino-containing biomolecules in the embryos. It is known that these anions have the capacity to strongly interact with DNA [27,75], aminoacids [71,72,73] and proteins [76,77,78] through hydrogen or dihydrogen and halogen bonds (C_c_-H···O and C_c_-H···H-B or N-H···H-B or B-I···H-N, respectively). The impact of halogen (F, Cl, Br, and I) insertion on the biological activity and isozyme selectivity have been reported to be a source of inspiration for medicinal chemists [79,80,81,82].

Therefore, with the aim to extract the retained cobaltabis(dicarbollides) from the respective eggs’ pellets, d_6_-acetone was added to these pellets that lost the yellow color after the acetone extraction. The yellowish d_6_-acetone solution under ^1^H{^11^B} and ^11^B{^1^H} NMR spectroscopy (Figures S14a and S15a,b) unambiguously confirmed the presence of the cobaltabis(dicarbollide) in the samples. As an example, the ^1^H{^11^B} spectrum of the treated embryos with Na[8,8′-I_2_-*o-*COSAN] displays a broad resonance at 4.41 ppm that corresponds to C_c_-H as well as the signals at 3.24, 3.07, 2.61, and 1.84 ppm corresponding to B-H resonances (Figure S15a), while its ^11^B{^1^H} spectrum fully overlaps with the one of the parent Na[8,8′-I_2_-*o-*COSAN] (Figure S14b). The ATR-IR spectra of the dried yellowish d_6_-acetone solution (Figure S15c) displayed the typical B-H band around 2.550 cm^−1^, which undoubtedly agrees with the presence of the anionic small metallacarborane anions in the dry eggs’ pellets before d_6_-acetone extraction.

In conclusion, Na[*o-*COSAN] and Na[8,8′-I_2_-*o-*COSAN] compounds were located in the *C. elegans’* embryos after being treated with a 200 µM solution of the compounds for 24 h at RT under gentle agitation. The small cobaltacarborane anions form hybrids (eggs-[*o-*COSAN]^−^ and eggs-[8,8′-I_2_-*o-*COSAN]^−^) with the chemical components of the embryos providing a yellowish color to the eggs. The hybrids are lightly soluble in water but were extracted with acetone. The *C. elegans* eggs’ development was affected by the hybrids’ formation between either Na[*o-*COSAN] or Na[8,8′-I_2_-*o-*COSAN] and the embryos’ chemical composition, be it DNA or proteins.

### 3.5. Cell Uptake Experiments

**Figure 10:**
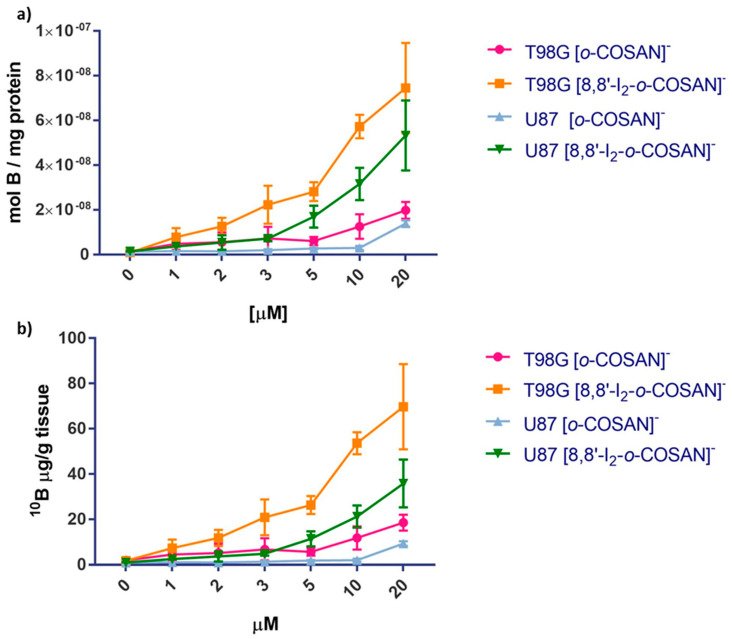
Upload of boron in U87 and T98G cells incubated in the presence of increasing concentration of [*o-*COSAN]^−^ and [I_2_-*o*-COSAN]^−^ for 24 h at 37 °C expressed as mol B/mg of protein (**a**) and µg ^10^B/g tissue (**b**). Error bars indicate the SD.

In order to assess whether the amount of boron taken up by target cells was enough to allow the set-up of an efficient BNCT procedure, U87 and T98G cells were incubated with increasing concentrations of [*o*-COSAN]^−^ and [8,8′-I_2_-*o*-COSAN]^−^, were analyzed for their boron content by ICP-MS technique. Both anions, [*o*-COSAN]^−^ and [8,8′-I_2_-*o*-COSAN]^−^, contain natural abundant boron; thus, the amount of ^10^B internalized was calculated as 19.9% of the total boron. Figure 10 shows that [*o*-COSAN]^−^ concentrations of 10 μM and 20 μM for U87 and T98G, respectively, in the culture medium were sufficient to load U87 with 2.7 and 12.9 μg/g of ^10^B and T98G with 11.8 and 18.5 μg/g of ^10^B. [8,8′-I_2_-*o*-COSAN]^−^ concentrations of 10 μM and 20 μM led to a higher loading for both cell lines, specifically of 29.5 and 49.8 μg/g of ^10^B for U87 and 53.6 and 69.7 μg/g of ^10^B for T98G. These quantities are well above the minimum boron threshold needed to perform BNCT. Under these experimental conditions, the cell viability measured by MTT was above 75% for all the cell lines.

From these results, we can conclude that the T98G cells, resistant to conventional radiotherapy, internalize significantly higher amounts of ^10^B with respect to U87 cells. This gives more importance to the use of BNCT as an alternative strategy for the treatment of this type of glioblastoma tumor.

### 3.6. BNCT Studies in Glioblastoma Cells

**Figure 11:**
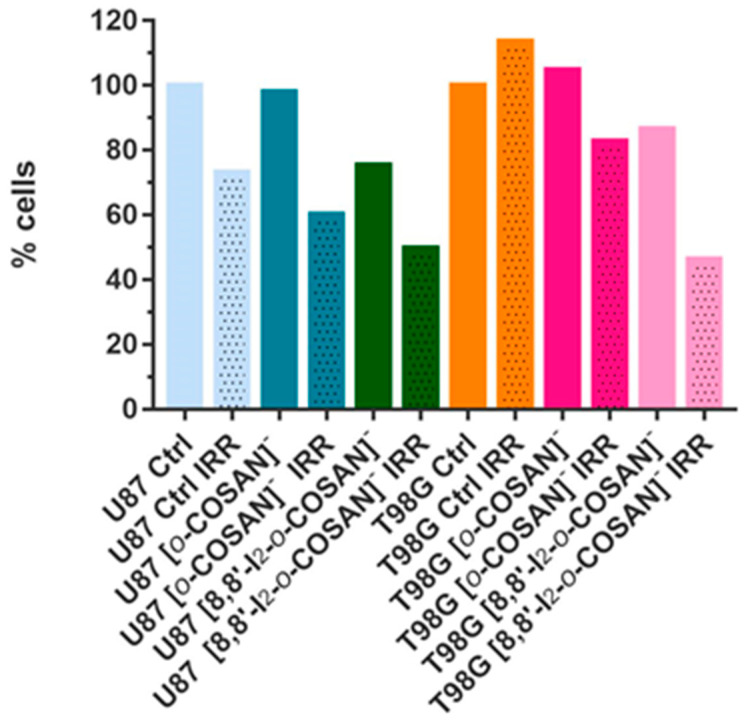
Percentage of viable U87 and T98G cells 24 h after neutron irradiation.

The viability of cells irradiated with thermal neutron treated ([*o*-COSAN]^−^-IRR and [8,8′-I_2_-*o*-COSAN]^−^-IRR) and not treated (CTRL-IRR) with Na[*o*-COSAN] and Na[8,8′-I_2_-*o*-COSAN] was evaluated and compared with the corresponding non-irradiated ones (i.e., CTRL, [*o*-COSAN]^−^ and [8,8′-I_2_-*o*-COSAN]^−^, respectively). Before irradiation, Na[*o*-COSAN] and Na[8,8′-I_2_-*o*-COSAN] 20 µM were incubated for 24 h at 37 °C with U87 and T98G cells. The irradiation time and reactor power were 15′ and 30 kW, respectively. Figure 11 shows the percentage of cells that survived neutron irradiation with respect to the non-irradiated control cells. From these results, it was evident that the T98G cells were more resistant to neutron irradiation, as no apparent cell-killing effect was observed for untreated cells. In contrast with Na[*o*-COSAN], the viability of T98G cells treated with Na[8,8′-I_2_-*o*-COSAN] was significantly reduced upon the irradiation treatment (Figure 11). As expected, the more pronounced effect of neutron irradiation was observed on these cells for the iodinated compound as a consequence of the higher amount of boron internalized (Figure 10). The U87 cells showed to be more sensitive to irradiation. In fact, a decrease of ca. 30% in the cell viability after irradiation was observed for untreated cells. For these cells, the trend was different; the Na[*o*-COSAN] presented a more promising profile for BNCT due to the cytotoxic behavior of its iodinated analog in these U87 cells.

Results showed that the T98G cells seemed to be more resistant to neutron irradiation, which is somehow in agreement with other previous reports [67,83].

To sum up, these studies unveil the potential of this type of molecules to be further evaluated as BNCT agents and may have a relevant role in eliminating glioblastoma cells resistant to other types of radiotherapies.

## 4. Conclusions

Glioblastoma (GBM) is a lethal primary brain tumor with a dismal prognosis having a high probability of recurrence. The failure of most prospective chemotherapeutic drugs in the preclinical evaluation stage may be related to the use of simplified two-dimensional (2D) in vitro models, which indicate that these models of GBM cannot translate the clinical scenario. Our studies indicated that only spheroids from the U87 cell line were found to have impaired growth after treatment with both compounds, suggesting an increased resistance from T98G spheroids, contrary to what is observed in the monolayer culture. Therefore, our results support the hypothesis that the 3D model seems to be a better representation of in vivo tumor microenvironment. Hence, the work highlights the need to employ 3D models for future GBM studies as it provides a more realistic model for cell-based studies. In vitro tests in U87 and T98G GBM cell lines conclude that the [*o*-COSAN]^−^ and [8,8′-I_2_-*o*-COSAN]^−^ at a non-cytotoxic concentration can load adequate ^10^B levels inside the cells for a successful BNC reaction. As expected, T98G cells, which are more resistant to standard radiotherapies, were also more resistant to neutron irradiation performed without boron addition. Interestingly, BNC reaction becomes more effective on T98G after their incubation with Na[8,8′-I_2_-*o*-COSAN] due to the higher amount of boron internalized by these cells, whereas no apparent cell-killing effect was observed for untreated cells. This observation demonstrates the potentiality of this alternative radiotherapy when the cell pre-targeting with boron-containing compounds happens successfully.

Subsequently, in vivo tests with both metallabis(dicarbollides) were performed in L4 *C. elegans* nematodes and their embryos. LD_50_ values for both cobaltabis(dicarbollides) in L4 *C. elegans* were found to be close to the IC_50_ determined for T98G in vitro after 72 h. These anionic small molecules, which are known to produce ion-pair complex with aminoacids and proteins by means of B-H···H-N dihydrogen bonds, could interact with the amine groups in the plethora of biomolecules of the organism and contribute to the toxicity. The in vivo studies of Na[*o-*COSAN] and Na[8,8′-I_2_-*o-*COSAN] with *C. elegans*’ embryos indicated that these molecules were located in the *C. elegans*’ eggs forming yellowish hybrids (eggs-[*o-*COSAN]^−^ and eggs-[8,8′-I_2_-*o-*COSAN]^−^) with the embryos’ chemical components. These hybrids, which are lightly soluble in water but dissolve in acetone, arrested the development of the larvae, probably due to the ion-pair complex formation with proteins.

Nevertheless, further in vivo evaluation in mammalian models, such as biodistribution studies in mouse models with GBM xenografts, are required to fully understand their ability to target the tumor cells, as well as to cross the blood–brain barrier.

To sum up, our results in vitro, in vivo, and irradiation experiments combined with their physicochemical properties strongly suggest that these anionic small molecules, in particular [8,8′-I_2_-*o*-COSAN]^−^, could be regarded as prospective candidates to be taken into account for future developments in the scope of BNCT, now that the facilities of accelerator-based neutron sources are more accessible providing an alternative treatment either alone or in combination with other conventional chemotherapeutics for resistant glioblastoma.

## Figures and Tables

**Table 1:** The chemical and physicochemical parameters of the two cobaltabis(dicarbollides), Na[*o*-COSAN] and Na[8,8′-I_2_-*o*-COSAN].

Parameters	[*o*-COSAN]^−^	[8,8′-I_2_-*o*-COSAN]^−^
Size (nm)	1.1 × 0.6	1.1 × 0.8
Molecular weight of the anion	323.75	575.54
Rotamer	Cisoid	Transoid
Intramolecular interactions	No	C_c_-H I
Intermolecular interactions in aqueous solution	Yes	Yes
Aggregates formation in H_2_O	Yes [34,51]	Yes [33,51]
DLS	Aggregates (d = 64 nm) in the range 1 < c < 29 mM. Aggregates (d = 1.4 nm) c > 29 mM.	Aggregates (d = 107 nm) in the range 1 < c < 110 mM. Aggregates (d = 1.1 nm) at c > 110 mM.
Solubility in H_2_O (mM)	1509 [32]	210 [32]
logS	3.18	2.32
Lipophilicity (P)	43.7	151.0
logP	1.64	2.18
E_1/2_ M^3+/2+^ (in V reference F_c_^+^/F_c_)	−2.10	−1.47

**Table 2:** IC_50_ (μM) of [*o*-COSAN]^−^ and [8,8′-I_2_-*o*-COSAN]^−^ towards U87 and T98G cells determined after 6, 24, 48, and 72 h of incubation. Data are mean ± SD.

Incubation Time	U87		T98G	
	[*o*-COSAN]^−^	[8,8′-I_2_-*o*-COSAN]^−^	[*o*-COSAN]^−^	[8,8′-I_2_-*o*-COSAN]^−^
6 h	222 ± 127	141 ± 85	368 ± 202	157 ± 84
24 h	186 ± 93	102 ± 62	148 ± 57	59 ± 17
48 h	127 ± 52	51 ± 15	57 ± 20	24 ± 10
72 h	84 ± 31	36 ± 15	14 ± 8	14 ± 6

**Table 3:** Concentrations of metallabis(dicarbollide) compounds tested for the viability assay.

Concentration (μM)	U87		T98G	
	[*o*-COSAN]^−^	[8,8′-I_2_-*o-*COSAN]^−^	[*o*-COSAN]^−^	[8,8′-I_2_-*o*-COSAN]^−^
<IC_50_	40	35	7	7
IC_50_ *	80	50	14	14
>IC_50_	100	100	19	19

* IC50 at 72 h determined by the MTT method.

## Data Availability

The data presented in this study are available in the main text and the Supplementary Materials.

## Supplementary Materials

The following are available online at www.mdpi.com/article/10.3390/cancers13246367/s1, Figures S1–S3: Representative images of the U87 and T98G spheroids before (day 3 of culture) and after 72 h (day 6 of culture) of exposure to the two compounds at above and below IC_50_ concentration, Figures S4 and S5: Characterization of the hybrids (eggs-control, eggs-[*o*-COSAN]^−^ and eggs-[8,8′-I_2_-*o*-COSAN]^−^) by optical microscopy, Figure S6: Photography of the *C. elegans* embryos after 24 h treatment. ATR-IR spectra, SEM/EDX of eggs-control, Figures S7–S11: eggs-[*o*-COSAN]^−^and eggs-[8,8′-I_2_-*o*-COSAN]^−^, Figures S12 and S13: UV-vis and ATR-IR spectra of D_2_O filtrated eggs-[*o*-COSAN]^−^and eggs-[8,8′-I_2_-*o*-COSAN]^−^, Figure S14: ^1^H{^11^B} NMR and ATR-IR spectra of d_6_-acetone filtrated after treatment with Na[*o*-COSAN], Figure S15: ^1^H{^11^B}, ^11^B{^1^H} NMR, and ATR-IR spectra of d_6_-acetone filtrated after treatment with Na[8,8′-I_2_-*o*-COSAN]. Scheme S1: *C. elegans* maintenance and structure, Scheme S2: the designed procedure for the study of new hybrids eggs-[*o*-COSAN]^−^and eggs-[8,8′-I_2_-*o*-COSAN]^−^.
