# Supplementary material for: Cobaltabis(dicarbollide) ([o-COSAN]−) as Multifunctional Chemotherapeutics: A Prospective Application in Boron Neutron Capture Therapy (BNCT) for Glioblastoma
Source: Cancers (Basel). 2021 Dec 19;13(24):6367. doi: 10.3390/cancers13246367 (PMC8699431; doi:10.3390/cancers13246367)
Supplement: Supplementary file 1 [file cancers-13-06367-s001.zip › cancers-1463700-supplementary.pdf]

cancers

MDPI

# Supplementary Materials: Cobaltabis(dicarbollide) ([*o*-COSAN]$^-$) as Multifunctional Chemotherapeutics: A Prospective Application in Boron Neutron Capture Therapy (BNCT) for Glioblastoma

**Miquel Nuez-Martinez, Catarina I. G. Pinto, Joana F. Guerreiro, Filipa Mendes, Fernanda Marques, Amanda Muñoz-Juan, Jewel Ann Maria Xavier, Anna Laromaine, Valeria Bitonto, Nicoletta Protti, Simonetta Geninatti Crich, Francesc Teixidor and Clara Viñas**

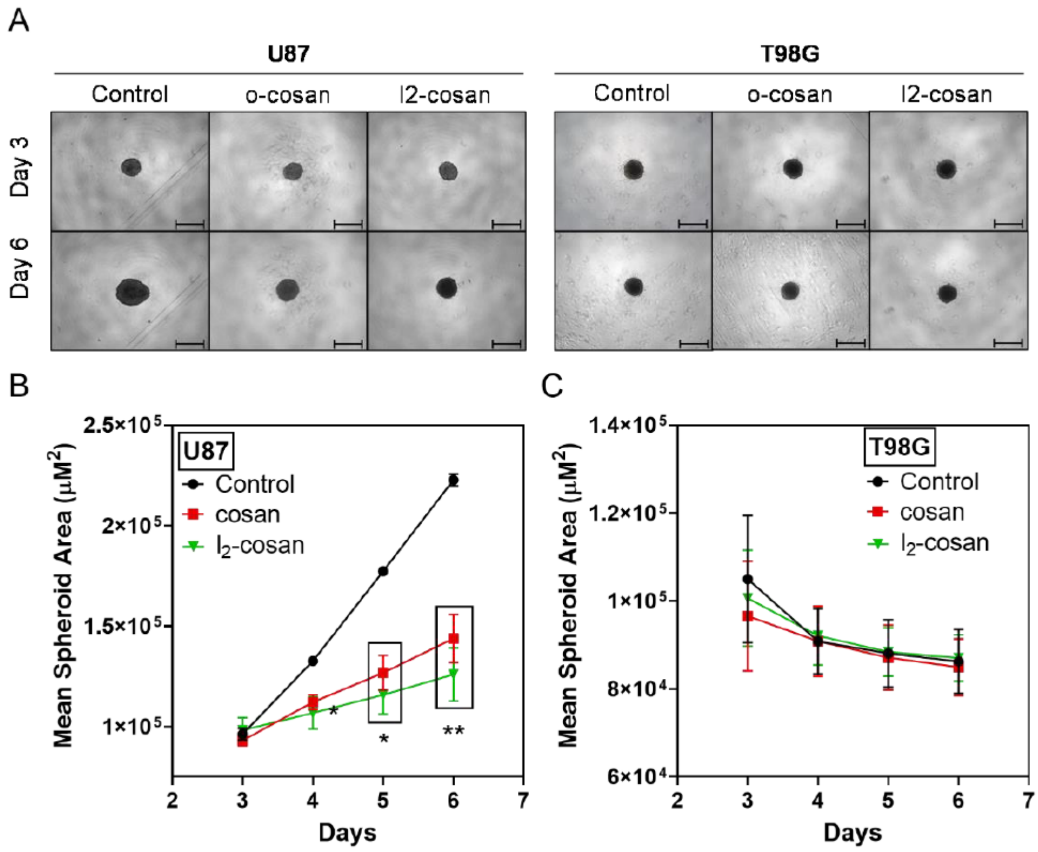


**Figure S1.** Effect of exposure to concentrations above the IC50 (determined by the MTT assay) of [*o*-COSAN]- and [8,8′-*o*-I2-COSAN]- on U87 and T98G spheroids. (**A**) Representative images of the spheroids before (day 3 of culture) and after 72 h (day 6 of culture) of incubation with the different complexes; (**B**) U87 and (**C**) T98G spheroids growth, represented by the mean spheroids area (in µM2) as a function of the number of days in culture. Controls consist of spheroids incubated only with medium. Data are presented as the average +/- SD of 3 independent assays for spheroids. Scale bars correspond to 500 µm. Statistical significance was calculated using one-way ANOVA, followed by Dunnett's test comparing treated spheroids/cells with control spheroids/cells (* $p \le 0.05$, ** $p \le 0.01$).

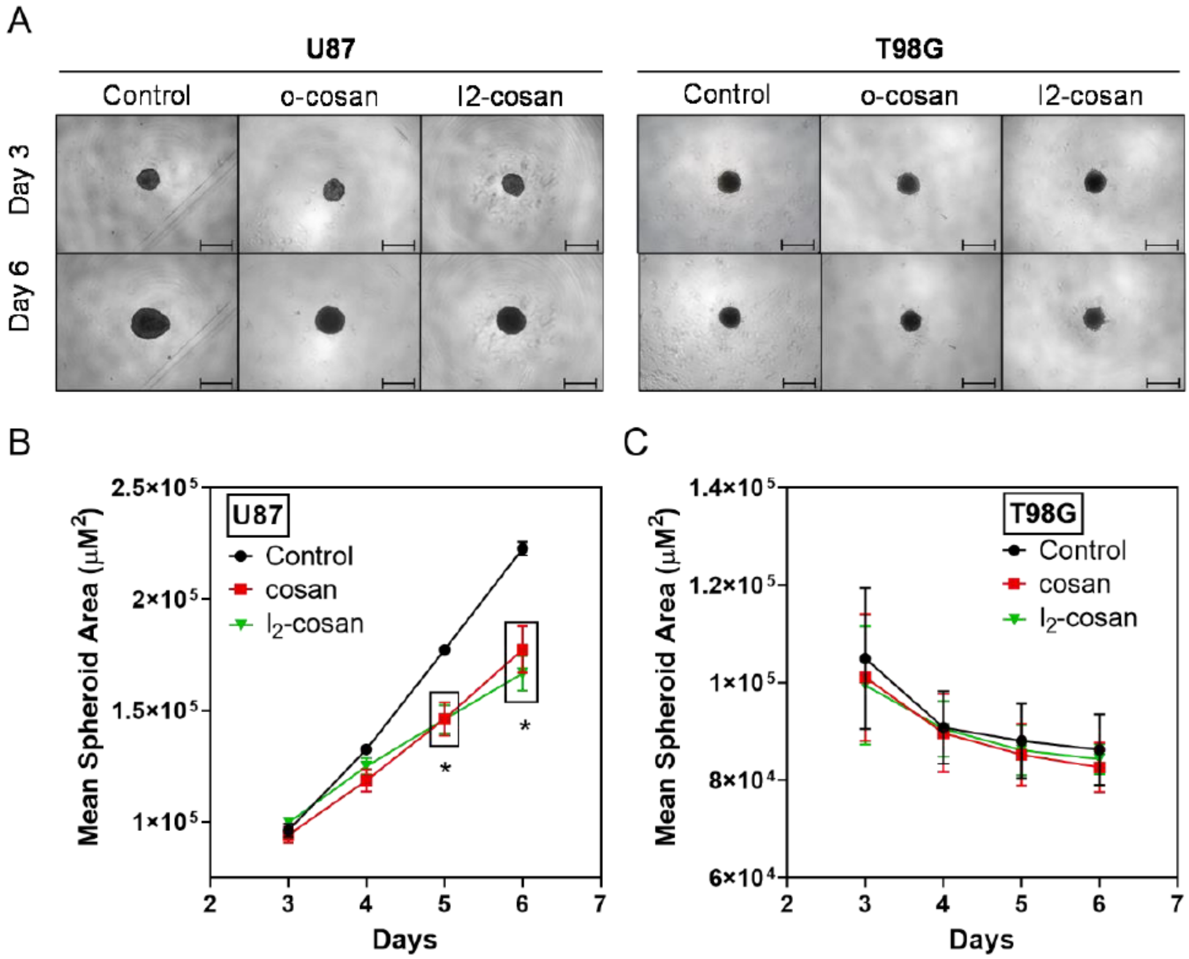


**Figure S2.** Effect of exposure to concentrations below the IC50 (determined by the MTT assay) of [*o*-COSAN]- and [8,8′-*o*-I2-COSAN]- on U87 and T98G spheroids. (**A**) Representative images of the spheroids before (day 3 of culture) and after 72 h (day 6 of culture) of incubation with the different complexes; (**B**) U87 and (**C**) T98G spheroids growth, represented by the mean spheroids area (in μM2) as a function of the number of days in culture. Controls consist of spheroids incubated only with medium. Data are presented as the average +/- SD of 3 independent assays for spheroids. Scale bars correspond to 500 μm. Statistical significance was calculated using one-way ANOVA, followed by Dunnett's test comparing treated spheroids/cells with control spheroids/cells (* $p \leq 0.05$).

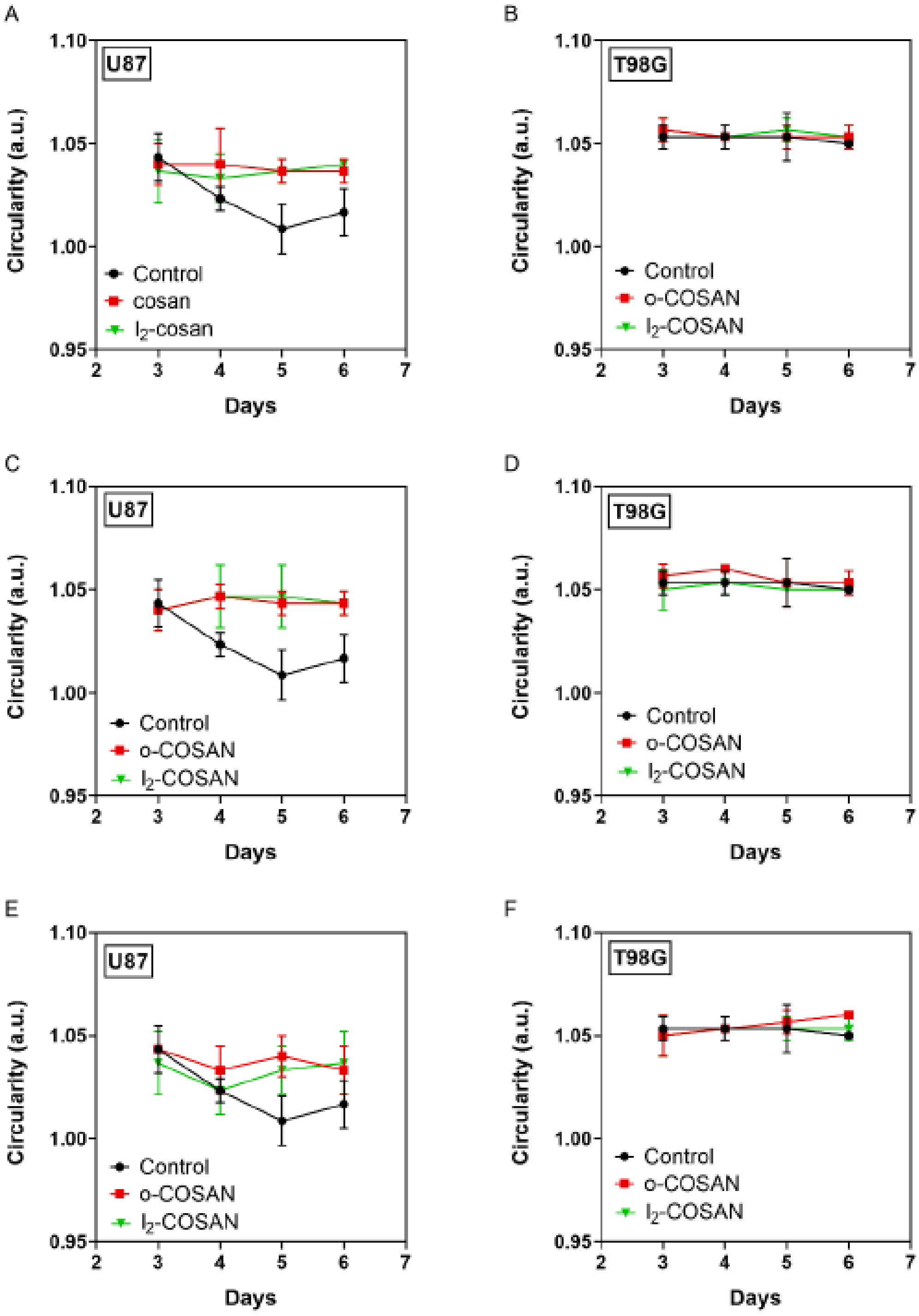


**Figure S3.** Effects of incubation of [*o*-COSAN]- and [8,8'-I2-*o*-COSAN]- on U87 (left panels) and T98G (right panels) spheroids' circularity. Effects of exposure to (**A**,**B**) 50. concentrations, (**C**,**D**) above IC50 concentrations, and (**E**,**F**) bellow IC50 concentrations on spheroids circularity, represented by the mean spheroids' circularity (in arbitrary units) as a function of the number of days in culture. Controls consist of spheroids incubated only with medium. Data are presented as the average +/- SD of 3 independent assays for spheroids. Statistical significance was calculated using one-way ANOVA, followed by Dunnett's test comparing treated spheroids/cells with control spheroids/cells.

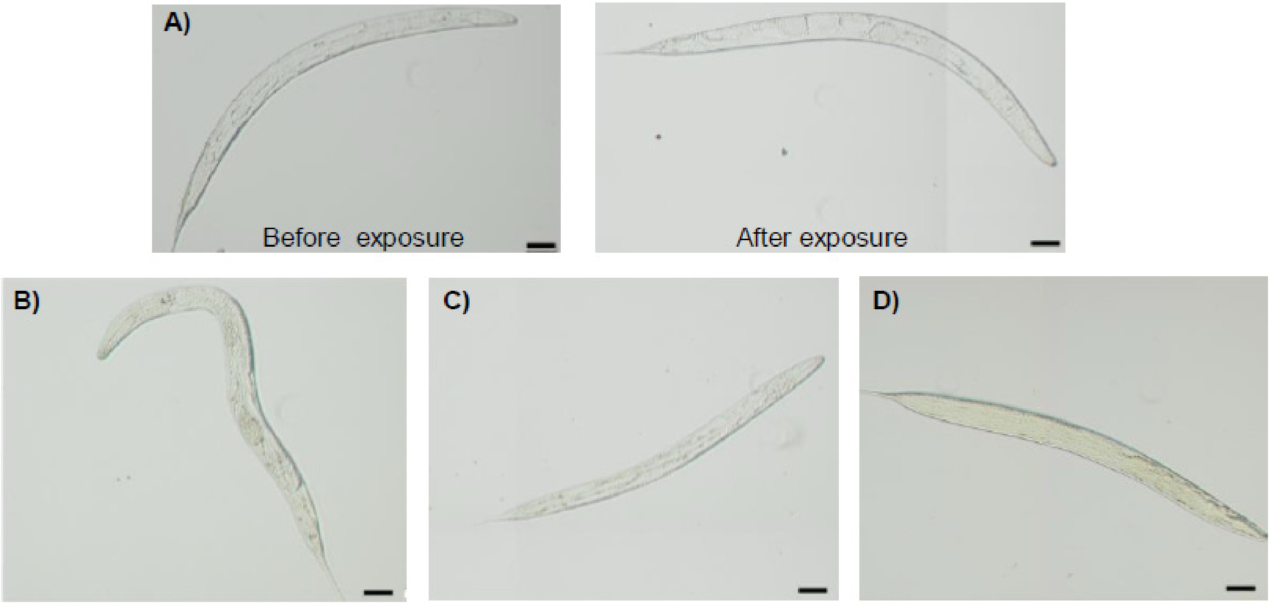


**Figure S4.** Optical microscopy images of L4 *C. elegans* L4 after 24h of incubation with different concentration of Na[*o*-COSAN]. (**A**) Control worms grow from L4 stage to the adult stage. (**B**) Worms incubated with 1µM [*o*-COSAN]- can develop till the adult stage. (**C**) At 10 µM [*o*-COSAN]- a high percentage of worms are dead in the L4 stage. (**D**) At 200 µM [*o*-COSAN]- worms are dead in the L4 stage with a yellowish color. Scale bar 50 µm.

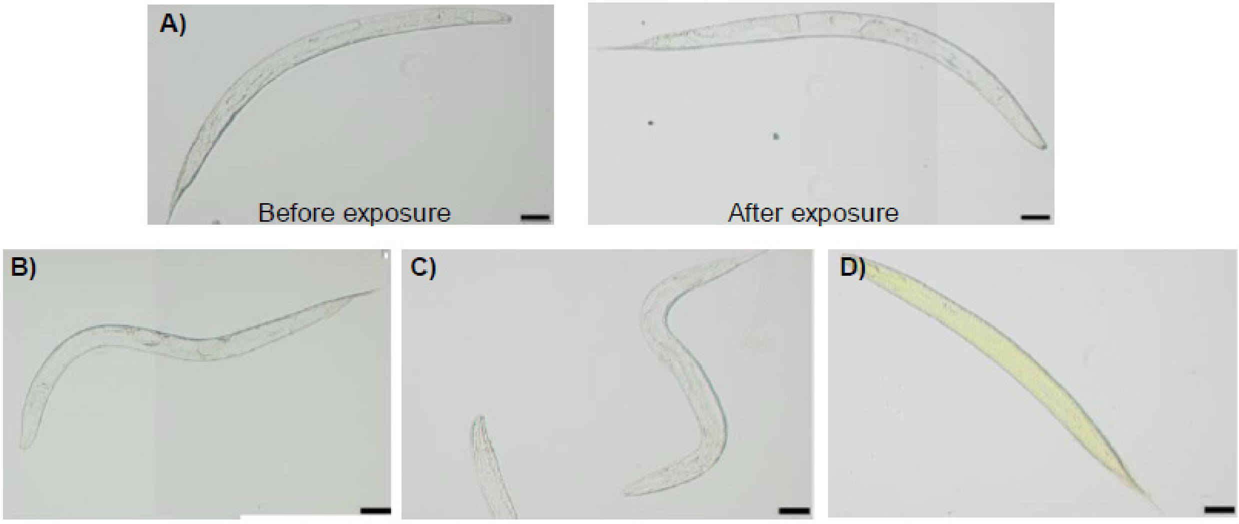


**Figure S5.** Optical microscopy images of L4 *C. elegans* after 24 h of incubation with different concentraTable 8. I2-*o*-CO-SAN]- . (**A**) Control worms grow from L4 stage to the adult stage. (**B**) Worms incubated with 1µM [8,8′-I2-*o*-COSAN]- can develop till the adult stage. (**C**) At 10 µM [8,8′-I2-*o*-COSAN] a high percentage of worms are dead in the L4 stage. (**D**) At 200 µM [8,8′-I2-*o*-COSAN]- worms are dead in the L4 stage with a yellowish color. Scale bar 50 µm.

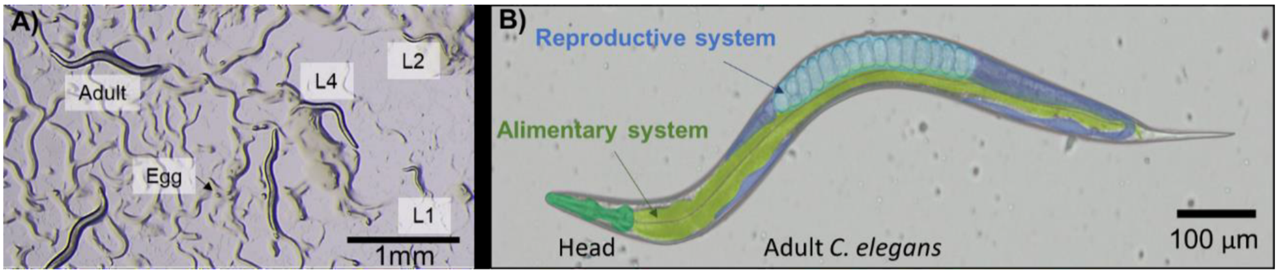


**Scheme 1.** *C. elegans* maintanance and structure. (**A**) Unsynchronized porpulation of *C. elegans* in NGM plates where different stages are labelled. (**B**) Anatomical image of an adult *C. elegans*.

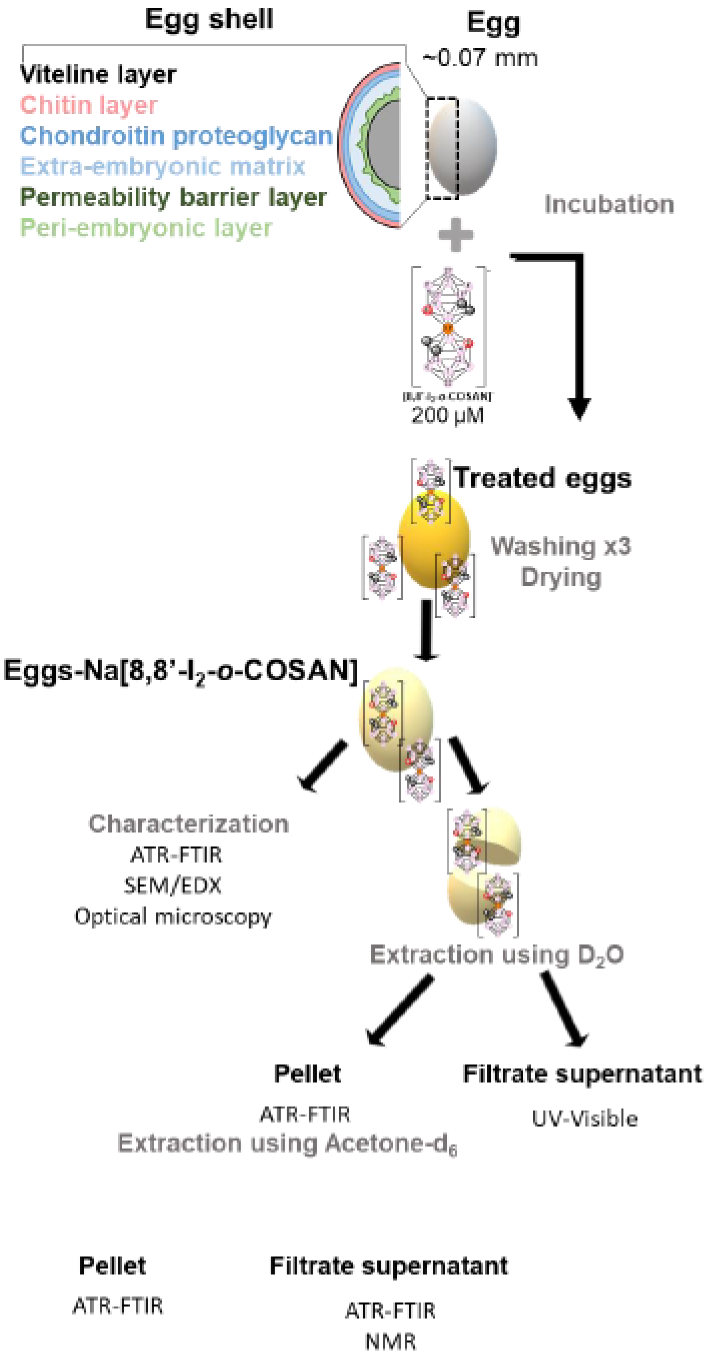


**Scheme 2.** Designed procedure for the study of formed new hybrids eggs/[8,8′-I2-*o*-COSAN]-.

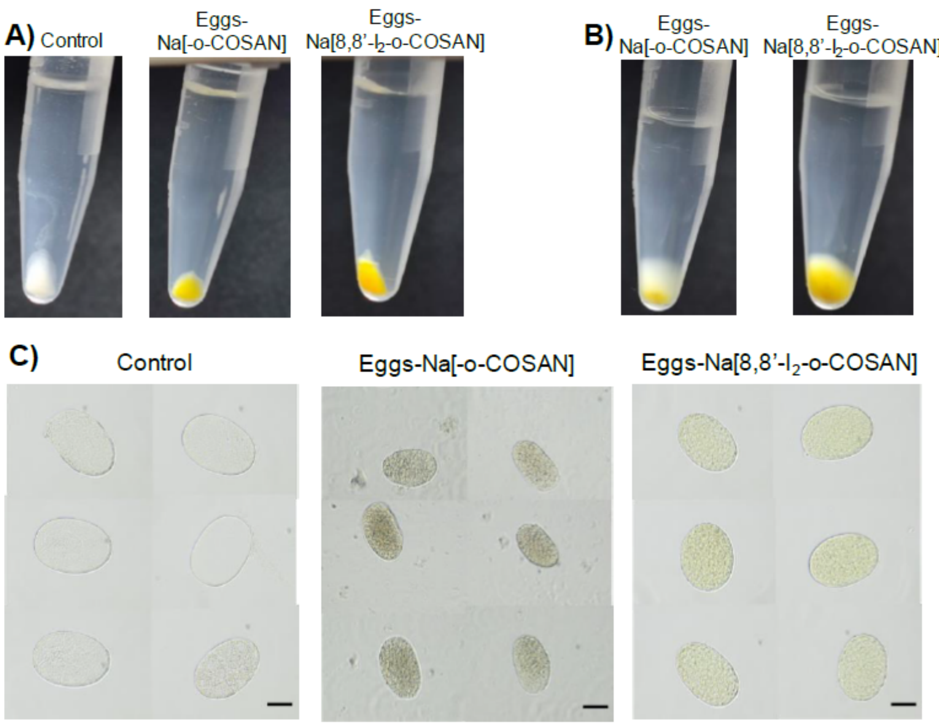


**Figure S6.** *C. elegans'* embryos treatment with cobaltabis(dicarbollides). (**A**) Photography of the *C. elegans* embryos samples after treatment for 24 hours at room temperature with 0 (control) and 200 M of Na[*o*-COSAN] and Na[8,8'-I2-*o*-COSAN]. (**B**) Photography of treated *C. elegans* embryos samples after cleaning three times with MQ water to remove the excess. (**C**)Their optical microscopy images. Scale bar: 20 µm.

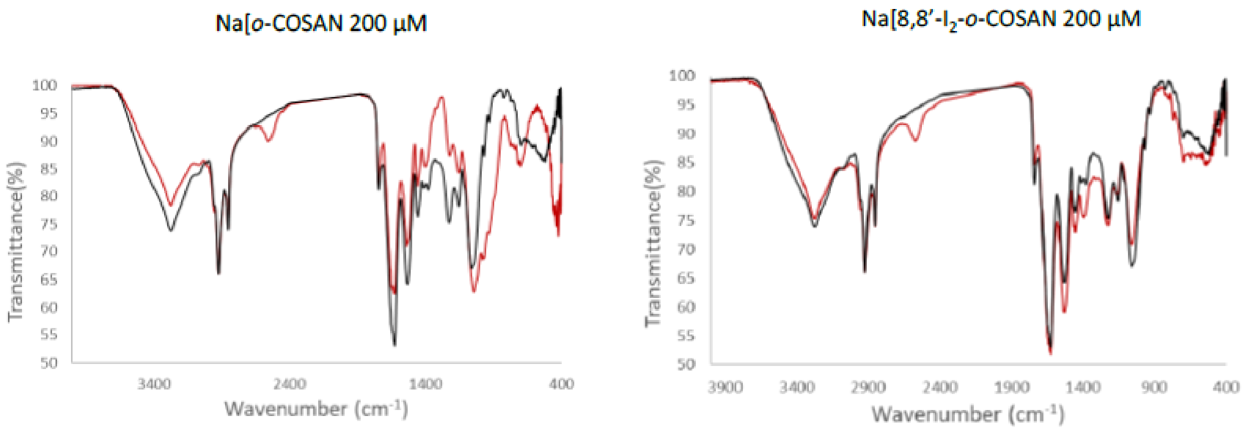


**Figure S7.** ATR-IR spectra of the *C. elegans* embryos samples after treatment for 24hours at room temperature with 0 (control) and 200 M of Na[*o*-COSAN] and Na[8,8'-I2-*o*-COSAN], washing (×3), centrifugated and dried at 60 °C for 17 hours. In black *C. elegans* embryos and in red *C. elegans* embryos after treatment with 200 M of cobaltabis(dicarbollide).

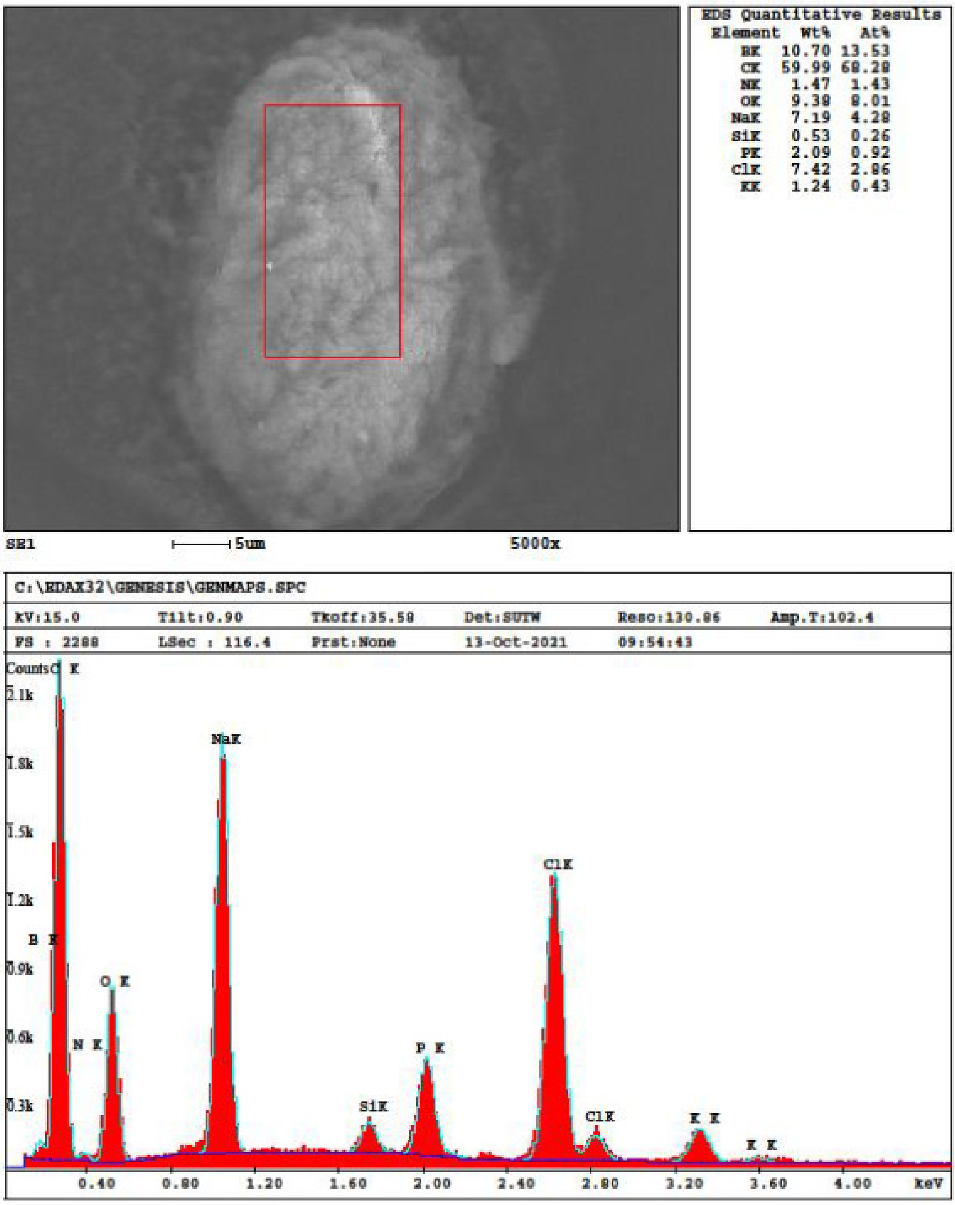


**Figure S8.** SEM/EDX of *C. Elegans* embryos control.

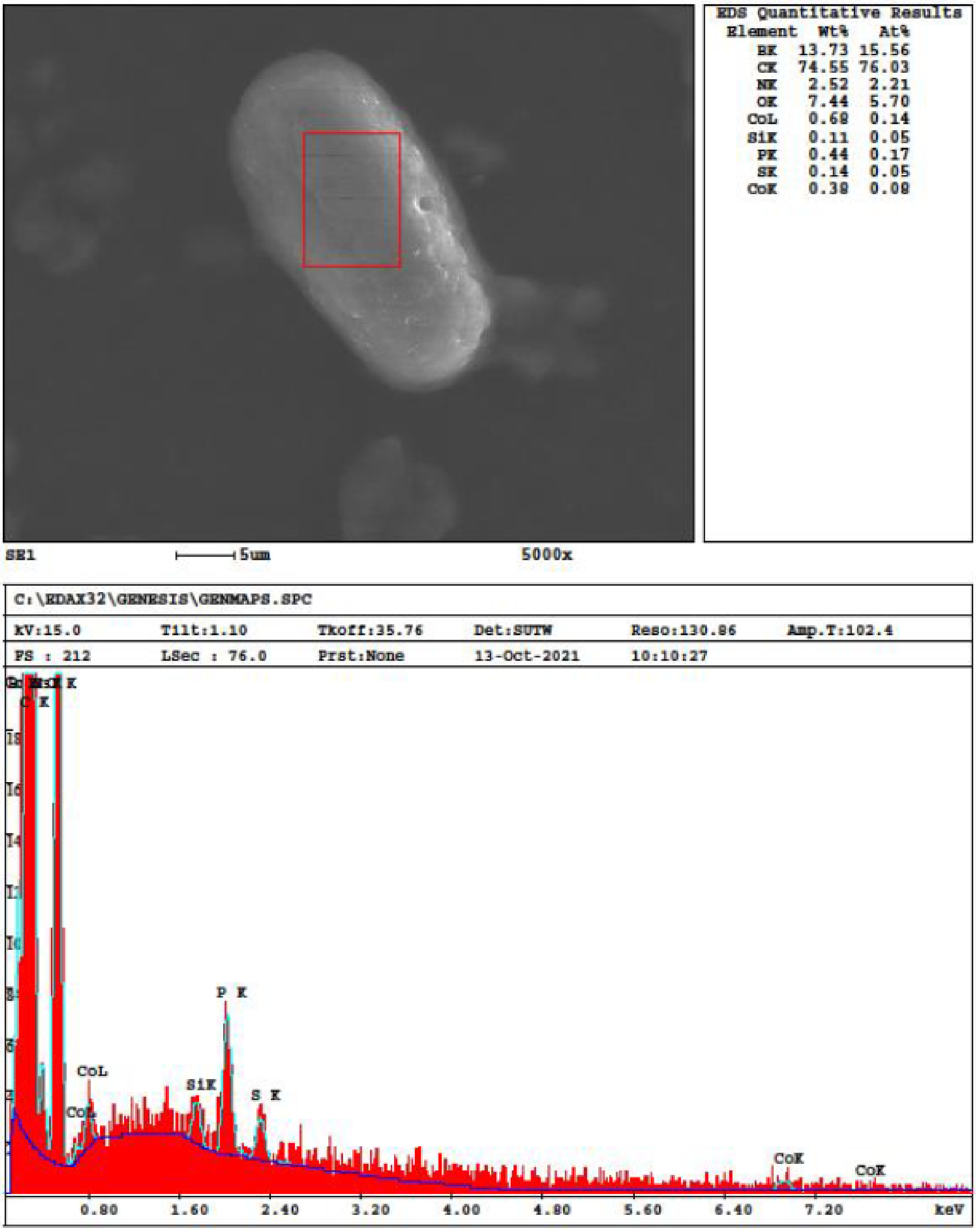


**Figure S9.** SEM/EDX of *C. Elegans* embryos after treatment with 200 M of Na[*o*- COSAN] for 24 h at room temperature under gently agitation.

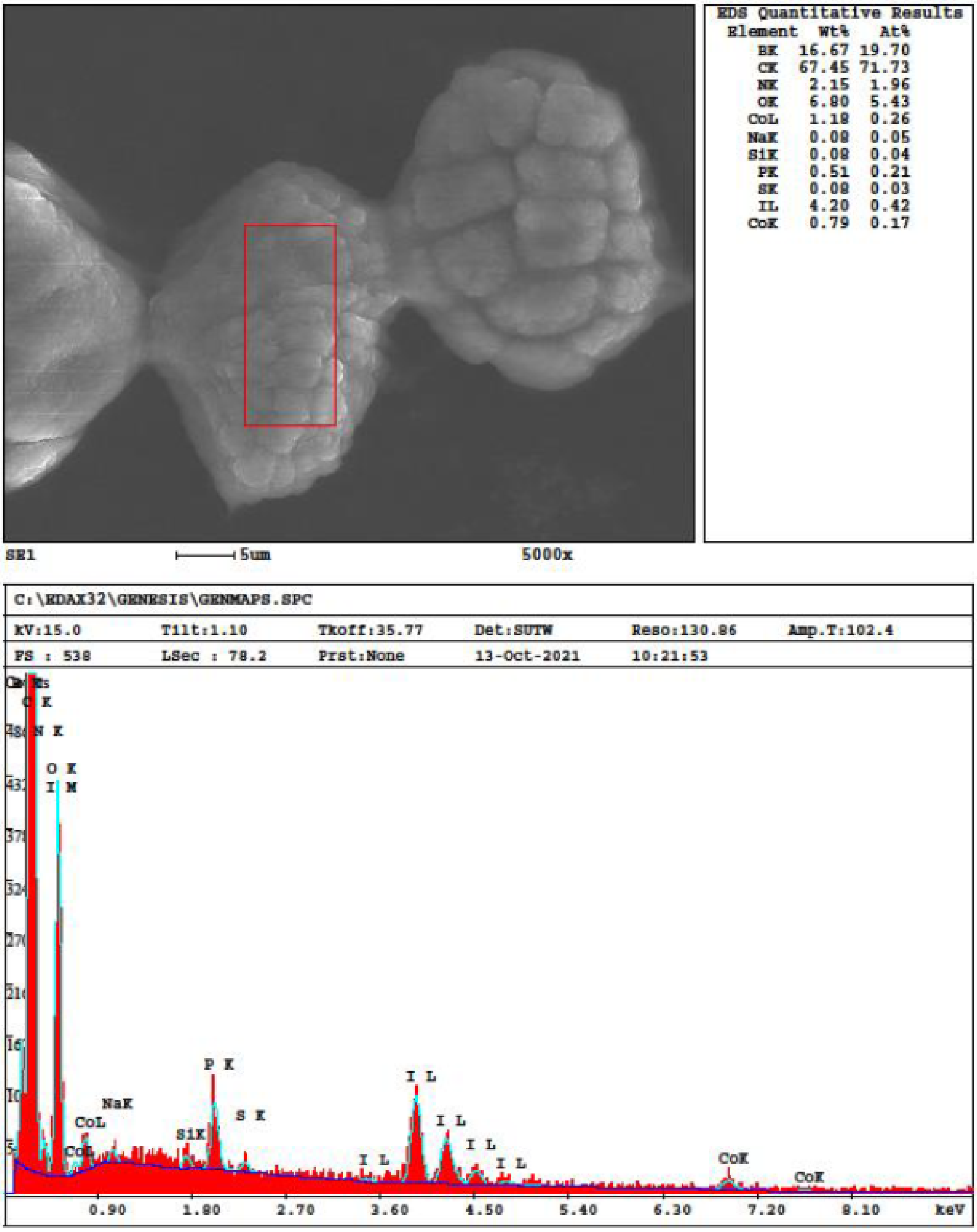


**Figure S10.** SEM/EDX of *C. Elegans* embryos after treatment with 200 M of Na[8,8′- I2-*o*-COSAN] for 24 h at room temperature under gently agitation.

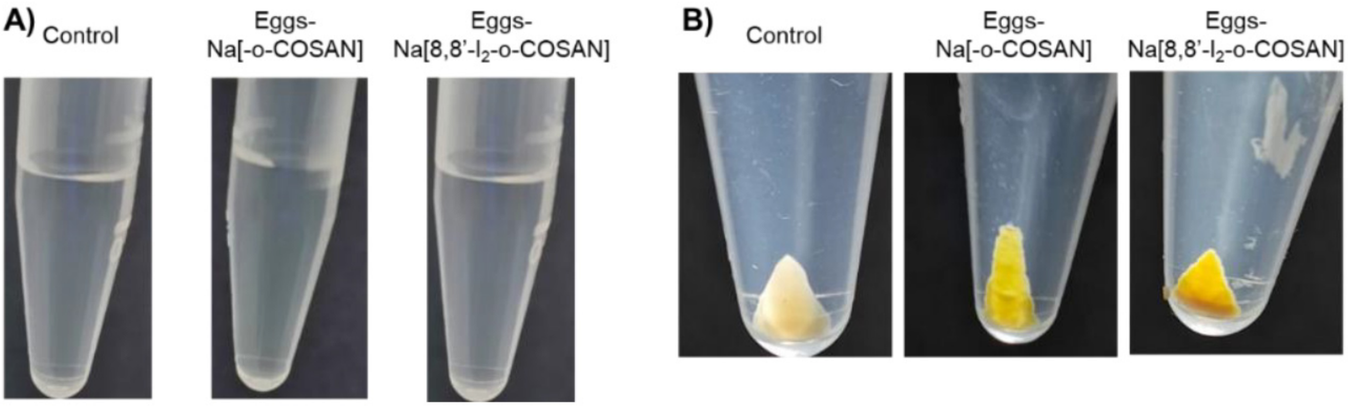


**Figure S11.** Cobaltabis(dicarbollide) extraction with D2O. a) Photography of the D2O filtrated supernatant solutions of the control and treated cobaltabis(dicarbollide) samples. b) Photography of the dried pellets of the control and treated cobaltabis(dicarbollide) samples after extraction with D2O.

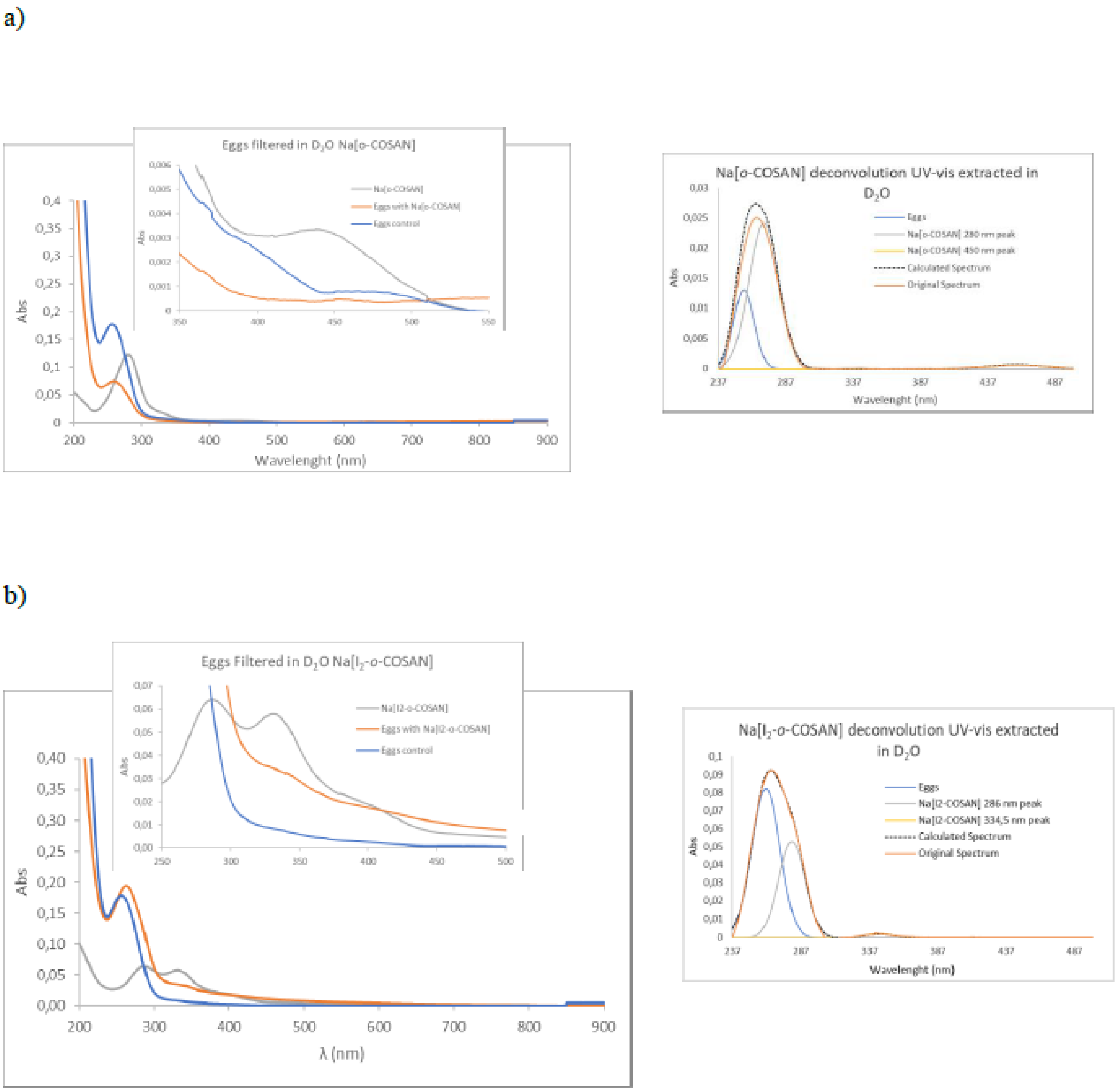


**Figure S12.** UV-*vis* spectra of D2O filtrated supernatant solutions. (**a**) After treatment with Na[*o*-COSAN]; inset a magnification of the spectrum and its deconvolution (right). (**b**) After treatment with Na[I2-*o*-COSAN]; inset a magnification of the spectrum and its deconvolution (right).

a)

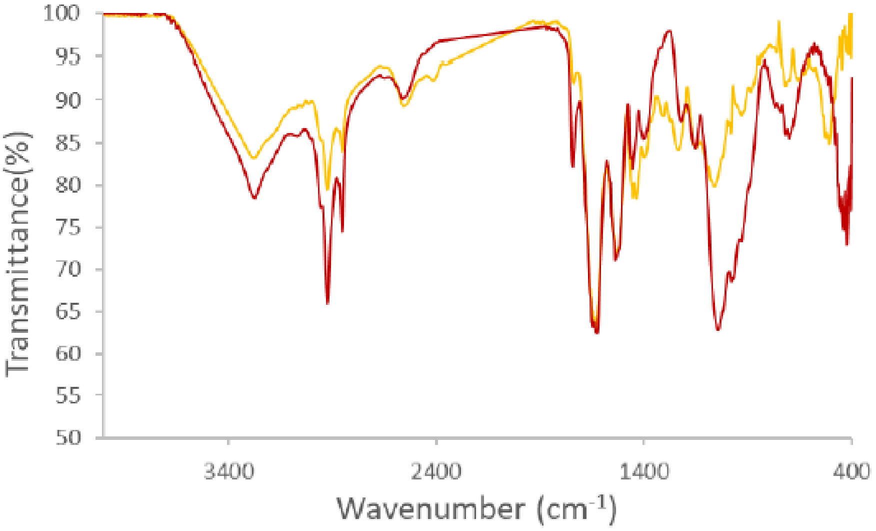


b)

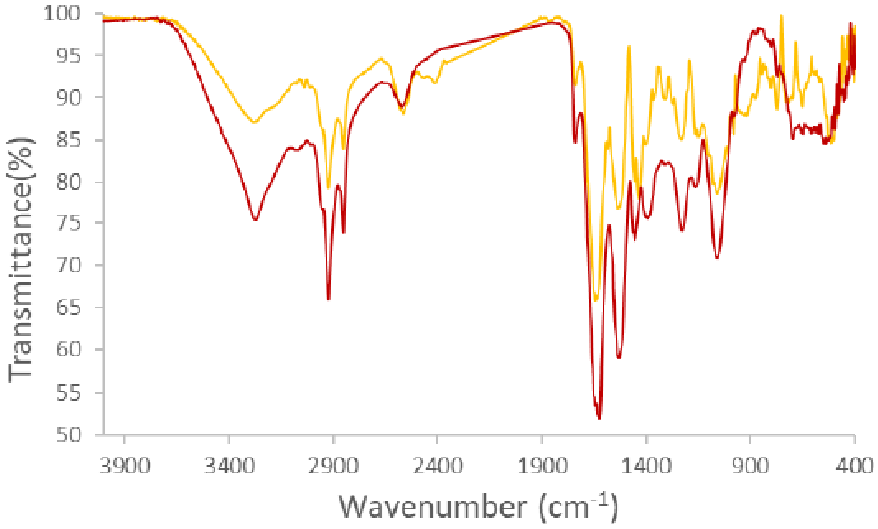


**Figure S13.** UV-vis spectra of D2O filtrated supernatant solutions. (**a**) After treatment with Na[o-COSAN]; inset a magnification of the spectrum and its deconvolution (right). (**b**) After treatment with Na[8,8-I2-o-COSAN]; inset a magnification of the spectrum and its deconvolution (right).

a)

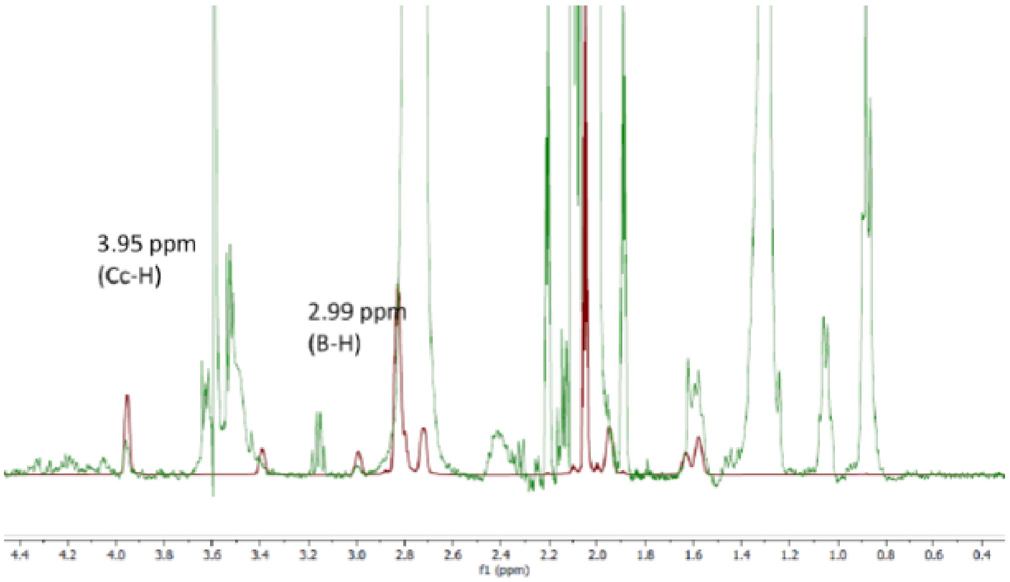


b)

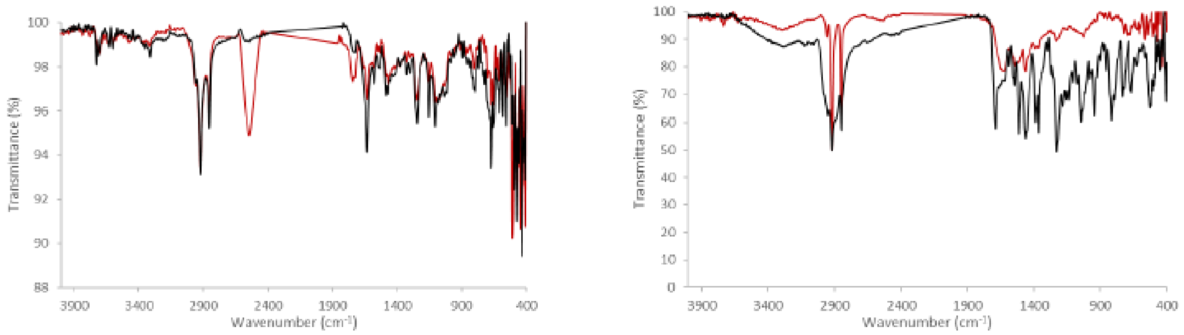


**Figure S14.** (**a**) 1H{11B} NMR spectrum of d6-acetone filtrated supernatant solution after treatment with Na[*o*-COSAN] in the range 4.5-0.4 ppm; *C. elegans* eggs with Na[*o*- COSAN] (green), Na[*o*-COSAN] (red). (**b**) Left, ATR-IR spectra of the dried d6-acetone filtrated supernatant solution after treatment with Na[*o*-COSAN] (red) and *C. elegans* eggs control (black); right, the residual pellet *C. elegans eggs* (red) and *C. elegans* eggs control (black).

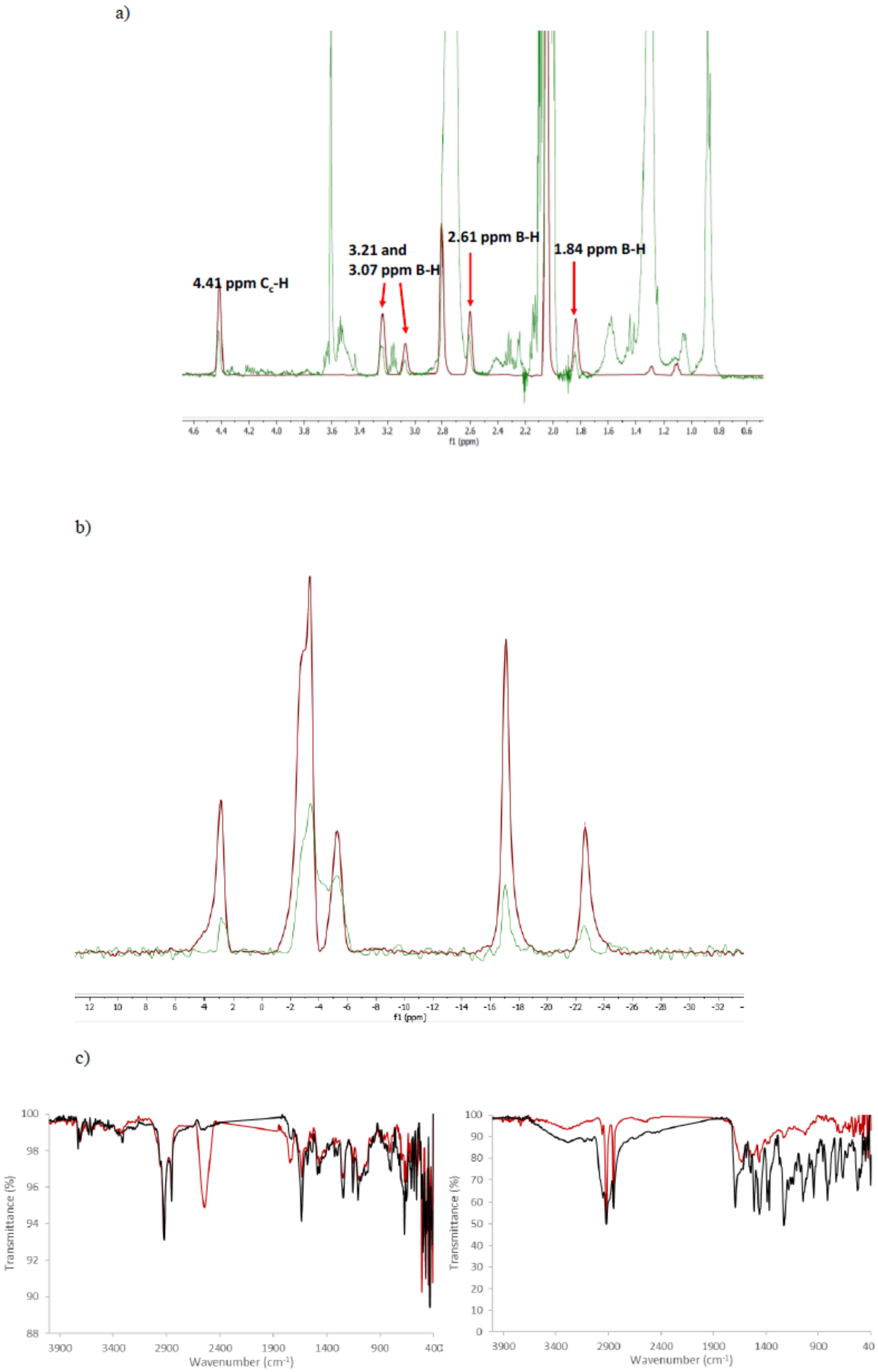


**Figure S15.** (**a**) 1H{11B} NMR spectrum of d6-acetone filtrated supernatant solution after treatment with Na[I2-*o*-COSAN] in the range 5-2 ppm; *C. elegans* eggs with Na[8,8′-I2-*o*-COSAN] (green), Na[8,8′-I2-*o*-COSAN] (red). (**b**) 11B{1H} NMR spectrum of a) d6-acetone filtrated supernatant solution after treatment with Na[8,8′-I2-*o*-COSAN] (red) and Na[8,8′-I2-*o*-COSAN] (green). (**c**) Left, ATR-IR spectra of the dried d6-acetone filtrated supernatant solution after treatment with Na[8,8′-I2-*o*-COSAN] (red) and *C. elegans* eggs control (black); right, the residual pellet *C. elegans eggs* (red) and *C. elegans* eggs control (black).
